# Probing the energy barriers and stages of membrane protein unfolding using solid-state NMR spectroscopy

**DOI:** 10.1126/sciadv.adm7907

**Published:** 2024-05-17

**Authors:** Peng Xiao, Philip Drewniak, Dylan Archer Dingwell, Leonid S. Brown, Vladimir Ladizhansky

**Affiliations:** Department of Physics and Biophysics Interdepartmental Group, University of Guelph, Guelph, ON N1G2W1, Canada.

## Abstract

Understanding how the amino acid sequence dictates protein structure and defines its stability is a fundamental problem in molecular biology. It is especially challenging for membrane proteins that reside in the complex environment of a lipid bilayer. Here, we obtain an atomic-level picture of the thermally induced unfolding of a membrane-embedded α-helical protein, human aquaporin 1, using solid-state nuclear magnetic resonance spectroscopy. Our data reveal the hierarchical two-step pathway that begins with unfolding of a structured extracellular loop and proceeds to an intermediate state with a native-like helical packing. In the second step, the transmembrane domain unravels as a single unit, resulting in a heterogeneous misfolded state with high helical content but with nonnative helical packing. Our results show the importance of loops for the kinetic stabilization of the whole membrane protein structure and support the three-stage membrane protein folding model.

## Teaser

An unfolding pathway of a membrane protein proceeds through two barriers and a native-like intermediate.

## INTRODUCTION

Folding of α-helical transmembrane (TM) proteins is commonly viewed in the context of two major stages (*1*). The first stage of insertion and helix formation is mediated by a translocon complex and is governed by the hydrophobicity of the TM fragments (*2*, *3*) or, from the energy perspective, by the membrane-partitioning free energy (*4*). Once TM fragments are inserted and helices are formed, their interactions occur within the lipid bilayer and result in formation of an α-helical bundle. In this second stage, optimization of interhelical interactions results in further reduction of free energy (*5*–*7*).

Pioneering experiments on bacteriorhodopsin have demonstrated that lateral interactions between TM helices drive the folding reaction, allowing the protein to reach a near native-like intermediate state (*8*–*12*) and creating an environment for the retinal cofactor binding stage, crucial for reaching the native state. The latter event is often described as the third stage of folding and, in addition to binding ligands and cofactors, also includes folding of loops, which further stabilizes the protein structure and completes its native tertiary fold (*13*). Recent unfolding studies by single-molecule force spectroscopy have yielded considerable additional insight into the second stage of folding process (*14*, *15*), allowing observation of transient intermediate states, determining thermodynamics and kinetics of unfolding, and, in favorable cases, probing unfolding and folding without pulling the protein out of the membrane (*16*–*19*).

In the majority of in vitro folding studies, membrane proteins are unfolded using chemical denaturants, e.g., detergents, and refolded back to their native state (*20*). As detergents have complex and destabilizing effects on membrane structure, which may distort folding/unfolding pathways, it is highly desirable to carry out unfolding and folding studies in lipids. Here, we use thermal denaturation to study unfolding of the lipid-embedded homotetrameric membrane protein human aquaporin 1 (hAQP1), without the use of detergent; this ensures that the unfolding process always occurs in a lipid environment, thereby accounting for the direct and indirect effects of the bilayer. We use a combination of hydrogen/deuterium exchange (HDX) and magic angle spinning (MAS) solid-state nuclear magnetic resonance (ssNMR) spectroscopy to determine key features of the energy landscape of hAQP1 and to characterize its unfolding pathway. HDX is a well-established technique in protein research, and it has been widely used in conjunction with solution NMR or mass spectrometry (MS) for probing both the thermodynamics and kinetics of protein folding (*21*–*26*). In recent years, ssNMR spectroscopy has also emerged as a powerful method for obtaining information on structural and dynamic properties of membrane proteins at the atomic level in lipid milieu (*27*).

**Fig. 1.**
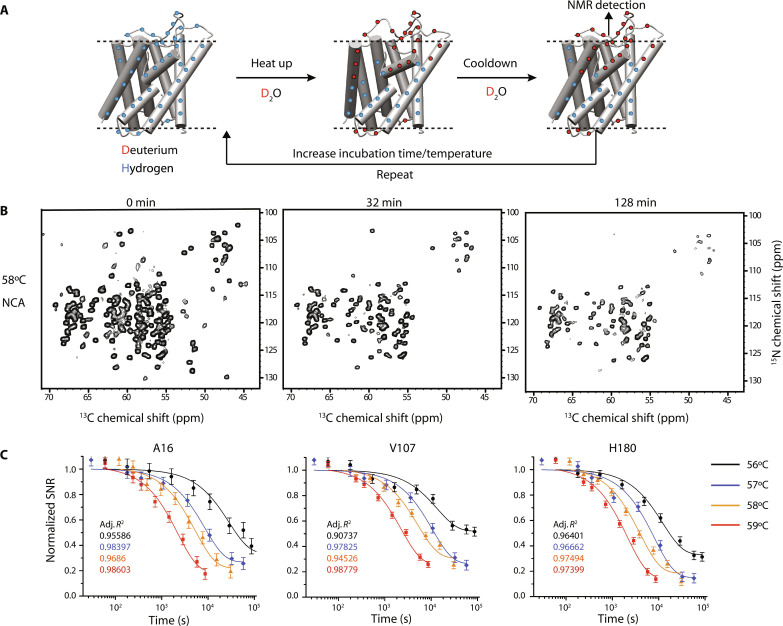
SSNMR detected HDX experiments for hAQP1. (**A**) General schematics of the ssNMR-detected HDX experiment on hAQP1. Dashed lines indicate a lipid bilayer. Protonated sites are in blue and deuterated sites are in red. The backbone amides in the TM region of a membrane-embedded protein are protected from solvent at the NMR experimental temperature (5°C). Higher temperatures increase the rate of unfolding and expose the TM core to D_2_O. Cooling the sample down to 5°C effectively seals up the protein stopping the exchange and preserving the HDX pattern generated during the incubation at high temperatures. This pattern is analyzed site-specifically by two-dimensional (2D) NMR. The scheme could be repeated at a fixed temperature as a function of the total incubation time, thereby reporting on the HDX rates, or at a fixed incubation time as a function of temperature, reporting on the progressive changes in the water-accessible surface. ppm, parts per million. (**B**) Representative 2D NCA spectra collected after incubation at 58°C at indicated total incubation times. (**C**) Representative HDX trajectories and their best mono-exponential fits for three TM residues at the four probed temperatures. Time axes are shown on a logarithmic scale. Errors are one SD.

In an hAQP1 monomer, six membrane-spanning alpha-helices H1-H6 are arranged into an hourglass-shaped structure with the aqueous channel in the center of the bundle (Fig. 1 and fig. S1). Two nonspanning half-helices HB and HE in the re-entrant loops B and E are packed end-to-end centrally as part of the channel. Both HB and HE feature highly conserved asparagine-proline-alanine (NPA) motifs located near the center of the pore where they connect to their respective helix-loop linkers (fig. S1B) (*28*–*31*). The NPA motifs along with the aromatic/arginine (ar/R) selectivity filter comprising residues F56, H180, C189, and R195 at the extracellular pore entrance (fig. S1B) are responsible for the water selectivity of hAQP1 (*32*–*34*).

The ~20-residue-long extracellular loop C in hAQP1 (fig. S1, A and B) is stabilized by a type II β-turn formed by a hydrogen bond between A130 and V133 (*35*, *36*), and this motif is conserved in other AQPs (table S1). The side chain of N127 in the middle of this loop adopts a buried conformation and interacts with the TM domain via a hydrogen bond with the side chain of S196 or with R195 and other residues in some structures (*30*, *37*–*39*), whereas the β-turn forms a hydrogen-bonded network with residues in the extracellular end of H5.

Our data point at the importance of loop C in stabilizing hAQP1 structure. Simultaneous residue-specific detection of multiple HDX rates in hAQP1, along with changes in the chemical shifts, allows for monitoring the kinetic stability of the native state and determining activation energies of unfolding while observing reversibility and, indirectly, structural changes within individual domains. We show that the breakdown of interactions within loop C and between the loop and the TM domain represents the first high-energy barrier step in hAQP1 unfolding; this leads to formation of an intermediate state, which retains nearly native interhelical contacts across many regions in the TM domain. In the second major step, the helical bundle unfolds irreversibly to the heterogeneous family of helical misfolded states. Our data support the idea of the third stage of folding in which the correct conformation of loop C kinetically stabilizes the native state of hAQP1 by raising the barrier of its unfolding.

## RESULTS

### Measurements of HDX

Temperature-dependent HDX rates report on protein conformational flexibility. Our methodology for measuring HDX rates is schematically depicted in Fig. 1A. It relies on the well-known fact that the TM core of a lipid-embedded multispanning helical membrane protein is protected from aqueous solvent under native conditions, and the backbone amides in solvent-inaccessible regions generally exchange on slow time scales (*40*–*43*). Thermal unfolding of hAQP1 immersed in D_2_O increases HDX rates in a manner that depends primarily on the unfolding rate as explained below. Cooling the sample back to low temperature (5°C) effectively quenches the H/D exchange and preserves the deuteration pattern generated at higher temperatures, so that the degree of D versus H substitution can be analyzed site-specifically using ssNMR. No appreciable exchange occurs at 5°C on the time scale of ssNMR experiments (20 to 30 hours), as was confirmed experimentally by comparing intensities of one-dimensional (1D) spectra collected before and after HDX-ssNMR detection.

To determine HDX rates, the high-temperature incubation/cooling/NMR detection cycle was performed repeatedly at increasing incubation times (Fig. 1A and see the “Thermal denaturation and H/D exchange experiments” section in the Supplementary Materials), and the extent of deuteration was quantified as a function of the total incubation time at a given incubation temperature. A complementary experiment was performed on a separate sample, in which the progression of HDX was probed site-specifically as a function of incubation temperature while keeping the incubation time constant, reporting on the temperature-dependent changes of the water-accessible surface of the protein (Fig. 1A).

### Site-specific detection of reversibility of domain unfolding

In addition to determining HDX rates and changes in water-accessible surface, we also monitored changes in carbon chemical shifts and linewidths after high-temperature incubation, as these parameters report on structural changes in the protein and reversibility of unfolding. In this series, we followed the same general scheme depicted in Fig. 1A and varied incubation temperature while keeping incubation time constant. Chemical shifts of aliphatic carbon atoms are sensitive structural reporters, whereas carbon linewidths are a measure of local structural homogeneity. Previous ssNMR studies of hAQP1 indicate a structurally homogeneous native state (*44*), without notable contributions from dynamics. High-temperature incubation and subsequent cooling to 5°C could produce two possible outcomes. If the protein returns to its original native conformation upon cooling (the case of a fully reversible transition), chemical shifts and linewidths in the carbon spectra remain unchanged. If a transition is not fully reversible and several protein conformations are produced, both chemical shifts and linewidths are expected to be affected. These changes could be detected site-specifically using 2D carbon-carbon correlation spectroscopy as discussed in the following, thereby allowing observation, at the atomic resolution, of both irreversibly unfolding regions and intact (or reversibly unfolding) domains within the same protein.

### Three different regimes of proton exchangeability

Differential scanning calorimetry (DSC) experiments carried out on the wild-type (WT) lipid-embedded hAQP1 show an unfolding transition at ~63°C with a pretransition onset starting at ~50°C (fig. S2A). Most lipids in the mixture used in this study (see lipids used in Supplementary Materials and Methods) contain at least one unsaturated fatty acid, thereby resulting in a low transition temperature. There is no lipid phase transition in the 30° to 75°C range (fig. S2A), ensuring that there is no interference with the hAQP1 unfolding transition.

HDX rates were measured at 56°, 57°, 58°, and 59°C in four individually prepared WT-hAQP1 samples. Each sample was repeatedly incubated in a D_2_O buffer at the target temperature for a given time interval and subsequently cooled down to 5°C for ssNMR detection after each incubation (see the “Thermal denaturation and H/D exchange experiments” section in the Supplementary Materials). 2D ^15^N-^13^Cα (NCA) and ^15^N-^13^CO (NCO) spectra with a short ^1^H/^15^N cross-polarization (CP) (*45*) excitation time of 300 μs were recorded after each incubation. Cross-peaks in these spectra primarily originate from excitation of amide protons, and their intensities report directly on the extent of protonation of the amide sites and are expected to weaken upon their progressive deuteration (Fig. 1, B and C, and figs. S3 and S4). Cumulatively, 138 backbone amide sites could be resolved in these experiments, in addition to the exchangeable amino groups of N127, R195, N122, Q137, N205, and the side chain of H180 (fig. S1A). The exchange rates *k*_HDX_ for backbone amides were extracted by fitting signal intensities of the resolved sites to a single exponential decay as a function of incubation time (Fig. 1C and fig. S4); more complex functions were used to fit the HDX trajectories of the NH_2_-bearing sidechains (see the “Data processing and analysis” section in the Supplementary Materials).

**Fig. 2.**
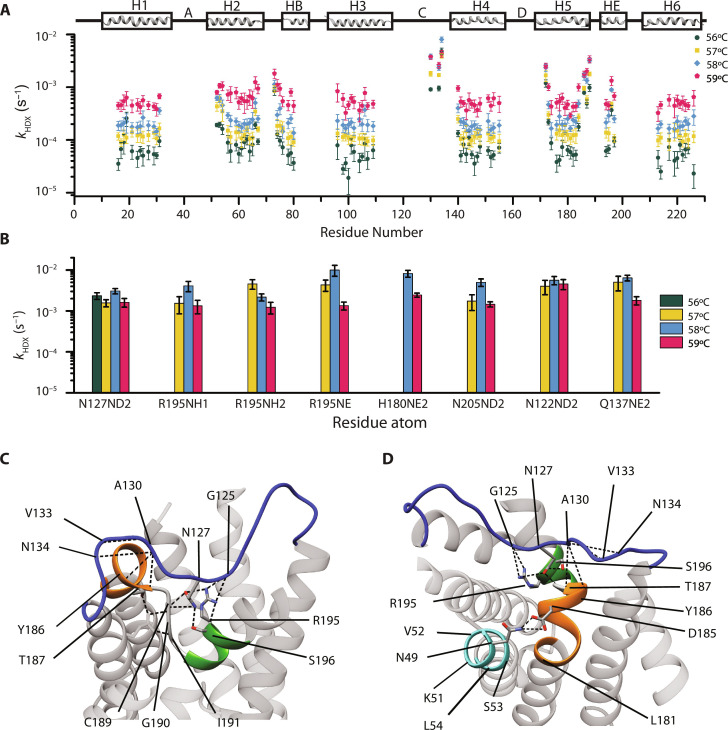
Hydrogen-deuterium exchange rates. (**A**) Site-specific exchange rates of backbone amides and (**B**) side chain groups. The rates are shown on a logarithmic scale. In (A), the secondary structure is shown on top. Errors are one SD. Only the rates that could be reliably determined are shown. (**C** to **D**) Cluster of interacting residues on the extracellular side that exchange in the intermediate regime in loop C (blue), helices H2 (cyan), H5 (orange), and nonspanning helix HE (green). Dashed lines represent hydrogen bonds. The homology model of hAQP1 is based on the high-resolution structure of bovine AQP1 [Protein Data Bank (PDB): 1J4N] constructed using SWISS-MODEL (https://swissmodel.expasy.org).

Three groups of residues with distinct exchange behavior were observed. Most residues in the first group belong to the TM domain, and they exchange slowly with typical rates of 10^−5^ to 10^−4^ s^−1^ at 56°C (Fig. 2A and table S2). The rates increase steeply, by approximately an order of magnitude at 59°C, but the exchange remains on a slow time scale.

Residues in the second group are mostly in the exposed loops. Their fast exchange at 56°C could not be quantified with the limited time resolution of our measurements (~20 s), but in contrast to the TM domain, their rates decrease with an increasing temperature (fig. S5). Similar behavior was observed by time-resolved HDX MS for exposed parts of globular soluble proteins (*46*), and it was ascribed to entropic solvent effects (*47*).

The third group includes atoms that exchange in the intermediate regime such as the backbone amides of A130, V133, N134, and the side chain of N127 in loop C, the backbone amide of S196 whose side chain forms a hydrogen bond with the side chain of N127, the side chain of R195 of the ar/R filter, which also interacts with loop C, and V52-L54 and Y186-C189 regions at the extracellular ends of helices H2 and H5, respectively (Fig. 2, A and B). These residues form a hydrogen-bonded network on the extracellular side (Fig. 2, C and D) with a higher degree of protection from the solvent than most exposed groups, yet they exchange much faster than the TM domains.

### Cooperativity of the exchange indicative of EX1 kinetics

Quantitative analysis of HDX for structurally protected sites is based on the widely accepted Linderstrøm-Lang model (*48*) described by a two-step reaction

$${\text{NH}}_{\text{closed}}\underset{k_{\text{cl}}}{\overset{k_{\text{op}}}{⇌}}{\text{NH}}_{\text{open}}\overset{k_{\text{ch}}}{\to }\text{ND}$$ (1)

Here, NH_open_, NH_closed_, and ND represent the open (exchange competent), the closed (exchange resistant), and the exchanged states of the NH groups in a protein, respectively. A conversion between the NH_open_ and NH_closed_ states is mediated by opening/closing conformational fluctuations with the rate constants *k*_op_ and *k*_cl_, respectively. Once the backbone amide is brought to the exchange-competent state, its proton exchanges for a deuteron at the intrinsic chemical exchange rate *k*_ch_, which is primarily determined by the protection factor from neighboring side chains and can be estimated at given pH and temperature (*49*).

In general, the observed exchange rates *k*_HDX_ depend on all the three rates *k*_op_, *k*_cl_, and *k*_ch_ and under the assumption that the protein is stable and *k*_op_ ≪ *k*_cl_, can be expressed as (*50*)

$$k_{\text{HDX}}\approx \frac{{k_{\text{op}}}^{}k_{\text{ch}}}{k_{\text{cl}}+k_{\text{ch}}}$$ (2)

There are two limiting conditions of HDX kinetics, depending on the relation between *k*_ch_ and *k*_cl_. The EX2 limit is commonly observed closer to the physiological conditions and is realized if *k*_ch_ ≪ *k*_cl_, leading to a simplified expression for *k*_HDX_

$$\text{EX}2:k_{\text{HDX}}\approx \frac{k_{\text{op}}}{k_{\text{cl}}}k_{\text{ch}}$$ (3)

In the EX2 regime, the observed rates depend on the chemical exchange rate *k*_ch_ which could vary by orders of magnitude depending on pH (*51*) and amino acid type (*49*).

The opposite EX1 regime occurs when *k*_cl_ ≪ *k*_ch_ and is often induced by a high concentration of denaturants, heat, or high pH (*52*–*55*). The EX1 kinetics is a direct measure of the rate of the opening transition and its interpretation does not require the knowledge of *k*_ch_

$$\text{EX}1:k_{\text{HDX}}\approx k_{\text{op}}$$ (4)

DSC measurements at pH 6, 7, and 8 indicate that hAQP1 stability depends on pH (fig. S2B), likely due to the presence of many titratable residues that are involved in crucial interactions responsible for the protein stability, e.g., R195, H180, K51, D185, E17, and E142 (fig. S1B). Similar pH-dependent stability was also reported for other membrane proteins (*56*–*61*). As this dependence makes it difficult to distinguish between EX1 and EX2 by measuring pH dependence of the exchange rates (*50*, *54*, *62*, *63*), we resorted to an indirect way of identifying EX1 regime through detecting correlated exchange events. In contrast to the EX2 regime in which the observed rates are expected to be strongly dependent on the amino acid type (*49*), the EX1 limit implies high cooperativity of exchange: all amide protons within a domain undergoing the same conformational fluctuation, e.g., unfolding, exchange at the same *k*_op_ rate (Eq. 4) (*54*, *62*, *64*, *65*). We detected two domains within hAQP1, which exchange in an internally cooperative manner. The first domain includes residues that exchange at intermediate rates and form an extensive spatially correlated cluster on the extracellular side (Fig. 2, C and D). First, the protected amino group of the N127 side chain is buried in the TM core and anchors loop C to the half-helix HE by forming hydrogen bonds with the side chain of S196, the side chain of R195 or both, and their exchangeable groups exhibit similar HDX rates (~10^−4^ to 10^−3^ s^−1^ at 56°C; Fig. 2, A and B, and tables S2 and S3). Second, both A130 and V133 in the type II β-turn exchange on the intermediate time scale, along with the backbone of N134. Furthermore, residues Y186 and T187, which participate in the conserved β-turn–H5 interaction between A130(N) and Y186(O)/T187(O) (Fig. 2D and table S2), exchange at rates similar to those determined for A130 and V133 (~10^−3^ s^−1^ at 56°C) (Fig. 2, A and B, and table S2) and are included in the same cluster.

Last, the extracellular ends of H2 and H5 are in close proximity, with intramonomer stabilizing interactions between D185 and N49 (*66*) (Fig. 2D) further substantiated by the directly detected short distance between N49Cγ and I184Cγ2 in the NMR spectra (*36*), and with intermonomer interactions involving D185 and K51 (*66*). Although the backbone amides of N49 and D185 exchange on a very fast time scale beyond the time resolution of our experiments, residues V52-L54 in helix H2, approximately one turn below N49 and deeper into the membrane, and I184 in H5 (next to D185), exchange much slower but still two to three times faster than the core residues of their respective helices (Fig. 2A and table S2). Thus, the extracellular end of H2 could be considered a part of the same cluster on the extracellular side.

The second domain involves most residues in the TM region, which exhibit correlated exchange at least an order of magnitude slower than the first domain. Similar exchange rates with similarly strong temperature dependence are observed in the core of all six TM helices at 57°, 58°, and 59°C (i.e., residues A16-G30 in H1, F56-Q65 in H2, I98-T109 in H3, I141-A155 in H4, V176-A183 in H5, and W213-I226 in H6) (Fig. 2A and table S2). Larger variations are observed within TM domains at 56°C; however, they are within experimental errors and there is still consistent correlated exchange within each helix. This cooperative exchange behavior at all probed temperatures is in agreement with the EX1 exchange kinetics. In the opposite case far from EX1, one would anticipate a much stronger dependence on the amino acid type, through the dependence of *k*_ch_ on the local protection factors.

### The hierarchy of activation energies suggesting a two-step unfolding process

The experimentally measured exchange rates *k*_HDX_ reflect the rates of domain unfolding, *k*_HDX_ ≈ *k*_op_ (*52*, *67*, *68*), and they are thus related to the activation energy *E*_a_ of unfolding by the Arrhenius equation

$$k_{\text{HDX}}\approx k_{\text{op}}=Ae^{-\frac{E_{a}}{\text{RT}}}$$ (5)

Here *A* is the pre-exponential factor, *T* is the absolute temperature, and *R* is the molar gas constant. The observed HDX rates *k*_HDX_ were fit to the natural logarithm of Eq. 5, $\text{ln}\left(k_{\text{HDX}}\right)\approx \left(-\frac{E_{a}}{R}\right)\left(\frac{1}{T}\right)+\text{ln}\left(A\right)$ as a function of inverse incubation temperature (1/*T*) to estimate *E*_a_ (fig. S6). In this approximation, both *A* and *E*_a_ are considered to be independent of temperature over a narrow range of 56° to ⁠59°C.

**Fig. 3.**
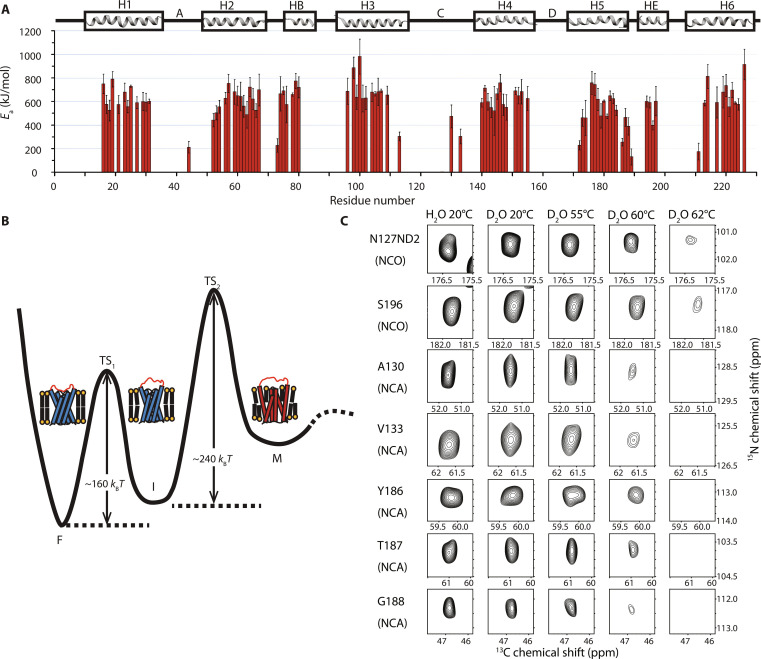
Proposed energy landscape of unfolding of hAQP1. (**A**) Site-specific activation energies of unfolding. Errors are shown as one SD. (**B**) Proposed unfolding energy landscape of hAQP1. The unfolding proceeds from the natively folded state F to an ensemble of misfolded states M through two transition barriers TS_1_ and TS_2_. The first barrier TS_1_ is ~300 to 400 kJ/mol (up to ~160 *k*_B_*T*) and corresponds to the unfolding of the extracellular cluster involving loop C and the interacting parts of helices HE, H5, and H2. The resulting intermediate state I retains many native interhelical contacts as indicated in the figure. The second unfolding transition from the I state over TS_2_ requires at least an additional ~600 kJ/mol (~240 *k*_B_*T*) and results in a helical state with an alternative interhelical packing as discussed in the text. Cartoon representations of helical bundles illustrate the proposed conformations corresponding to the F, I, and M states. (**C**) Progression of H/D exchange as a function of temperature for selected residues in the extracellular cluster: side chain of N127 and backbones of A130 and V133 from loop C, S196 from HE, and Y186-G188 in H5 and H5-HE linker. Incubation conditions and the types of experiments (NCA/NCO) are indicated. All spectra were collected on the same sample. The first contour level is at five times root mean square of the spectral noise.

Residue-specific activation energies are shown in Fig. 3A and could be classified into two groups. The first group with *E*_a_ of ~300 to ⁠400 kJ/mol (120 to ⁠160 *k*_B_*T*) includes the same extracellular cluster of interacting residues in loop C and in the nearby helices HE, H2, and H5, which show a cooperative intermediate exchange behavior: S196 (HE), which interacts with Nδ2 of N127; the hydrogen-bonded pair A130-V133, which stabilizes the β-turn in loop C; the fragment Y186-G188 in the extracellular end of H5, which interacts with A130(N) via Y186(O) or T187(O) (Fig. 2, C and D); and the exposed residues of H2, which interact with Y186-G188 via intra- and intermonomer contacts with D185 (Fig. 2D). Most residues in the TM region with activation energies of ~600 kJ/mol (~240 *k*_B_*T*) or higher constitute the second group. It should be noted that, although we estimate and report residue-specific *E*_a_ values, they represent the barriers that account for the collective unfolding motions within large domains, e.g., the spatially connected cluster of extracellular residues or major segments of TM helices stabilized by helix-helix interactions.

On the basis of the analysis of activation energies, we propose a two-step model of hAQP1 unfolding (Fig. 3B). The first step occurs over the barrier TS_1_ of ~300 to 400 kJ/mol (~160 *k*_B_*T*) and involves destabilization of loop C, initially through breakage of the hydrogen bond between V133(N) and A130(O), followed by breakage of the bond between Nδ2 of N127 and S196 side chain. Although not resolved in the activation energy plot, this proposed sequence of events is further justified with additional measurements discussed in the next section. The unfolding of loop C occurs concurrently with the destabilization of interactions between this loop and the fragment Y186-G188 of H5 and propagates further to the end of H2. The second unfolding step (Fig. 3B) requires additional energy to disrupt interhelical interactions within the TM domain and corresponds to melting of the helical bundle, crossing the second high-energy barrier TS_2_ (~240 *k*_B_*T*) and leading to an ensemble of misfolded states, which is characterized by a nonnative interhelical packing as discussed in the following.

Within this model, loop C and, specifically, the intra-loop V133(N)-A130(O) and loop-helix N127Nδ2-S196 hydrogen bonds play a key role in stabilizing the overall fold of hAQP1. To confirm the importance of these interactions, we have performed additional temperature-dependent Fourier transform infrared (FTIR) experiments on the N127A and V133P mutants, in which these hydrogen bonds are disrupted, using WT-hAQP1 as a reference (see the “Temperature-dependent ATR-FTIR experiments” section in the Supplementary Materials). Broadened amide I bands in the FTIR spectra of both N127A and V133P mutants collected in H_2_O at room temperature indicate that along with high helical content, there is also noticeable nonhelical structure that is not present in the WT-hAQP1 (fig. S7A), suggesting that both mutants are partially misfolded. This is further supported by the enhanced exchangeability of the mutants: While ~55% of backbone amides, mostly in loops and tails, exchange at room temperature in the WT-hAQP1, incubation of both mutants in D_2_O results in an ~80% loss of the amide II band intensity (reflecting backbone C-N-H vibrations), thereby suggesting that the TM core in both mutants is substantially more exposed to a polar solvent than in the WT (fig. S7, B to E). Furthermore, nearly complete HDX in the mutants occurs at 40° to 50°C, whereas a similar extent of exchange in the WT is observed at 65°C (fig. S7, B to E).

### Conformation of the unfolding intermediate and misfolded states

To gain further insight into the sequence of the unfolding events and to investigate structure of the unfolding intermediate and misfolded states, we carried out additional temperature-dependent HDX-ssNMR and FTIR studies. The same methodology as the one depicted in Fig. 1A was followed, except that the measurements were conducted as a function of temperature while the incubation time at high temperatures was kept constant at 2 min. For the HDX-ssNMR measurements, a WT-hAQP1 sample was incubated in D_2_O at progressively increasing temperatures of 20°, 55°, 60°, 62°, and 64°C (see the “Thermal denaturation and H/D exchange experiments” section in the Supplementary Materials), cooled down to 5°C, and subjected to ssNMR analysis after each incubation. In comparison to the HDX rates measurements, discussed above, in this temperature series, we could probe unfolding and limits of its reversibility closer to the unfolding transition up to 64°C. 2D NCA and NCO spectra were collected to detect the progression of H/D exchange and temperature-dependent changes in the water-accessible surface. These measurements were complemented with the measurement of 2D ^13^C-^13^C correlation spectra, which primarily originate from excitation from nonexchangeable protons; these spectra are not sensitive to the H/D exchange but may contain structural signatures of the trapped intermediate or misfolded states. Incubation at high temperatures close to the unfolding transition produces a population of partially unfolded states. Upon cooling, this population would either return to the folded (F) state in the case of reversible unfolding or will be trapped in the intermediate (I) or misfolded (M) state. The former scenario is realized if the thermal unfolding reaction does not cross the energy barrier TS_1_ so that the majority of partially unfolded hAQP1 molecules populate energy states close to the energy minimum corresponding to the F state (Fig. 3B). However, if TS_1_ or TS_2_ are crossed, a substantial population of partially unfolded or misfolded states may be generated, and these states may be trapped upon cooling. Under this scenario, unaltered 2D ^13^C-^13^C spectra would indicate fully reversible unfolding, whereas considerable spectral changes such as shifting or disappearance of peaks or line broadening would reflect the presence of the trapped I or M states and indirectly, on their respective conformations.

For FTIR measurements, a separately prepared sample was incubated in H_2_O (to avoid isotopic shifts due to the exchange) at 25°, 40°, 50°, 55°, 60°, and 65°C, and cooled down to room temperature after each incubation to record FTIR spectra (see the “Temperature-dependent ATR-FTIR experiments” section in the Supplementary Materials). Similar to ssNMR, spectroscopic signatures reporting on the secondary structure of the trapped I and M states would be present in the FTIR spectra, provided that a large population of these states is produced irreversibly during the high-temperature incubation and trapped upon cooling.

The preserved amide I and II bands in the FTIR spectra of the WT-hAQP1 sample (fig. S7F) indicate that the secondary structure is unchanged throughout the entire temperature series and in particular after incubations at 60° and 65°C, which are expected to produce irreversibly trapped intermediate and misfolded states. Thus, we can conclude that the protein retains its helicity even in the misfolded state, as known for other membrane proteins.

Data from ssNMR experiments add further detail to this picture. At lower temperatures, cross-peak intensities corresponding to residues within the extracellular cluster involving loop C (A130, V133, Nδ2 of N127), S196, and the end of helix H5 (Y186-G188) in the NCA and NCO spectra show no noticeable exchange, which indicates that this cluster remain protected from solvent (Fig. 3C). Conserved positions and linewidths of cross-peaks in the 2D ^13^C-^13^C correlation spectra indicate that the protein remains natively folded and structurally homogeneous up to the incubation point of 55°C (fig. S8, A to C). Thermal unfolding is either minor and does not produce detectable populations of the intermediate/misfolded states or is largely reversible upon cooling.

Incubation at 60°C results in nearly complete exchange of A130 and V133 amides (Fig. 3C), thereby suggesting destabilization and partial unfolding of the backbone of the β-turn in loop C. The exchange progression for these two residues correlates with that for the backbone amides of T187 and G188 of H5, suggesting the breakage of the β-turn–H5 interaction. However, the side chain of N127 and the backbone amide of S196 remain protected at 60°C (Fig. 3C), thereby indicating that loop C remains anchored to the TM domain and unfolds at a later stage. Moderate broadening of 2D ^13^C- ^13^C correlation spectra collected after the incubation at 60°C (fig. S8D) indicates partially irreversible unfolding but the line broadening effects remain local and mostly limited to the residues at solvent-exposed regions.

Simultaneous exchange of N127 sidechain and the amides of S196 and Y186 after an additional incubation at 62°C further confirms the continuing unfolding of loop C and the end of H5 (Fig. 3C). This occurs concomitantly with a substantial broadening and disappearance of many peaks in the carbon spectra (fig. S8E), suggesting that the TS_1_ barrier has been crossed, and the spectrum after incubation at 62°C no longer represents the pure native state but contains noticeable contributions from the trapped unfolding intermediate state.

Further incubation at 64°C results in an almost complete disappearance of the detectable peaks in the carbon-carbon correlation spectrum due to the strong line broadening effects across the entire protein (fig. S8F), thereby indicating a structurally heterogeneous ensemble of misfolded states. This ensemble is, however, helical, as evident from the preserved amide I and II bands in the FTIR spectra of the WT-hAQP1 sample after incubation at 65°C (fig. S7F); it possibly corresponds to a bundle with altered interhelical packings as schematically illustrated in Fig. 3B.

### Unfolding intermediate with native-like intramonomer core and intermonomer interface

**Fig. 4.**
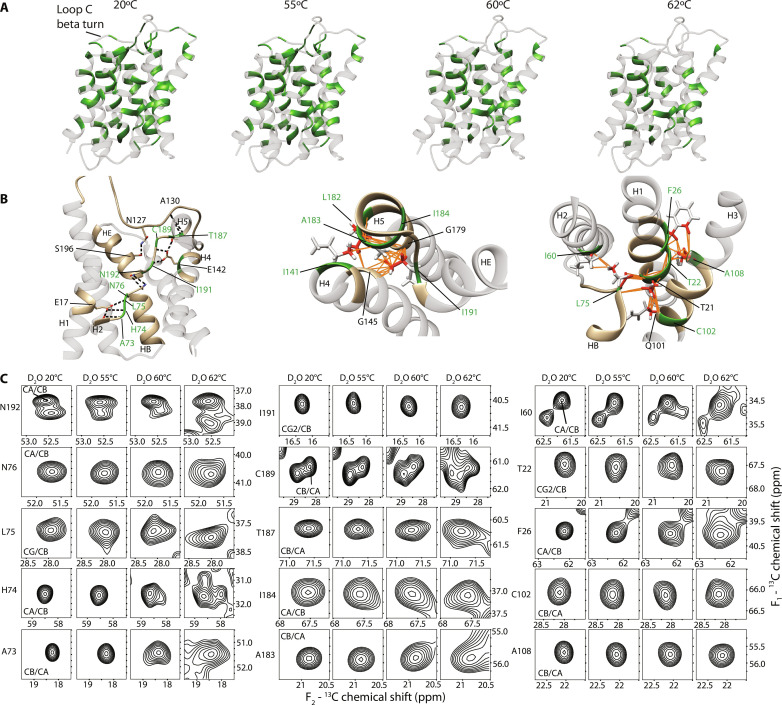
Conformation of the unfolding intermediate. (**A**) hAQP1 structure with residues having conserved chemical shifts after incubations at indicated temperatures shown in green. (**B**) Interacting clusters involving residues with conserved chemical shifts. Left: Highlight of residues in the re-entrant loop connecting H2 and HB, the re-entrant loop connecting H5 and HE, and in helices H1 and H4; middle: highlight of residues in helices H4 and H5 and the re-entrant loop connecting H5 and HE; right: highlight of residues in helices H1, H2, H3, and part of the re-entrant loop connecting H2 and HB. Hydrogen bonds are indicated by dashed lines. Atoms that are involved in favorable intramonomer van der Waals packing are colored red and their contacts are indicated by orange lines. Residues whose peaks are shown in (**C**) are shown in green. NMR-refined structure of hAQP1 (PDB: 6POJ) was used. (C) Selected cross-peaks from the 2D ^13^C-^13^C spectra, corresponding to residues with conserved chemical shifts that are involved in native intramonomer contacts preserved at higher temperatures. The first contour level in each panel is at half of the maximum peak intensity.

**Fig. 5.**
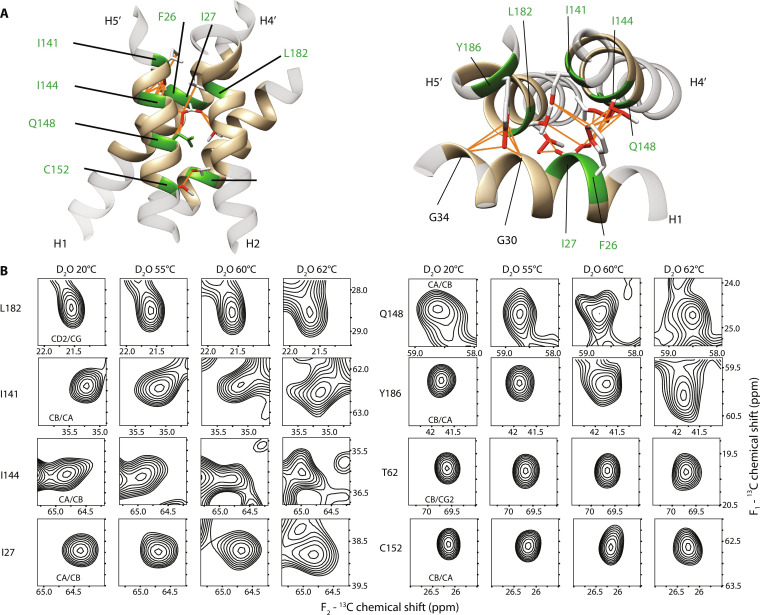
Intermonomer interface is resistant to thermal denaturation. (**A**) Side view (left) and top view (right) of an intermonomer interface. Atoms that are involved in van der Waals interactions are shown in red, and their contacts are indicated by orange lines. Residues shown in green correspond to cross-peaks shown in (**B**) and in Fig. 4C. The homology model based on PDB: 1J4N was used in this analysis. (B) Selected cross-peaks from the 2D ^13^C-^13^C spectra at indicated temperatures. The first contour level in each panel is at half of the maximum peak intensity.

Despite the overall spectral broadening observed after incubations at 60° and 62°C, there are many remaining resolved peaks in the spectra that are either sharp or only moderately broadened (Figs. 4 and 5 and fig. S8). The samples after these incubations are a mixture of the folded, the intermediate, and possibly the misfolded states. As the chemical shifts and intensities of the resolved peaks remain unchanged (Figs. 4 and 5 fig. S9), they, therefore, represent residues that experience similar structural environment in the folded and intermediate states. From the analysis of these peaks, we can therefore infer the conformation of the intermediate state.

The residues with resolved peaks form a nearly continuous network extending across the helical bundle to the intermonomer interface (Fig. 4A). Conservation of the interhelical contacts between these residues leads us to conclude that the intermediate state has a native-like interhelical packing. Within the monomer, the chemical shifts and linewidths of the backbone correlations of N76 and N192 of the highly conserved NPA motifs in HB and HE half-helices are largely preserved; L75 and I191 next to the NPA motifs give rise to sharp cross-peaks with linewidths comparable to those of N76 and N192; cross-peaks of the neighboring H74 and A73 and C189 and T187 in the loops connecting HB-H2 and HE-H5 toward the cytoplasmic and extracellular sides, respectively, remain sharp up to 60°C and only modestly broaden at 62°C (Fig. 4, B, left, and 4C). The network extends further on the extracellular side from I191 to helix H5: I191 fits tightly in the pocket formed by G145 (H4) and G179 (H5) and is also in a close contact with the side chain of A183 in H5, which is one turn above G179 (Fig. 4, B, middle, and C). A183 and the neighboring I184 produce sharp peaks at temperatures up to 60°C and are only moderately broadened at 62°C (Fig. 4C). In this region, A183 (H5) is also in contact with I141 (H4) and next to L182 (H5), both of which display conserved shifts and linewidths as well, and the latter two residues also participate in the intermonomer packing discussed below (Fig. 5, A, left, and B).

On the cytoplasmic side, the stable core extends from HB to H1-H3 through L75, which is flanked by the side chains of T21 (H1), I60 (H2), and Q101 (H3); the cross-peaks of I60, T21 and the neighboring T22, and of C102, which is next to Q101, are either sharp or moderately broadened (e.g., I60) up to 62°C (Fig. 4, B, right, and C). The side chain of T22 is involved in a van der Waals packing network with the side chains of A108 in H3 and F26 in H1 (Fig. 4C), whose corresponding peaks remain sharp or moderately broadened. Notably, the latter residue contributes to the intermonomer packing discussed below.

In addition to identifying the native-like intramonomer core of the unfolding intermediate, the cross-peak patterns also point to a stable intermonomer interface, thereby suggesting that the intermediate state also retains native-like quaternary structure. In the native state, the intermonomer interface of hAQP1 is stabilized by numerous van der Waals interactions between the H1 and H2 and H4′ and H5′ pairs of helices of two adjacent monomers [Fig. 5A, left; analyzed with UCSF Chimera (*69*)]. The network of residues with conserved shifts and linewidths extends from the cytoplasmic side of the aqueous pore to F26 (H1) and from the extracellular side of the pore to I141 (H4′) and L182 (H5′), all of which are involved in van der Waals interactions at the intermonomer interface (Fig. 5, A, left, and B). F26 (H1) interacts with I141 and I144 of H4′ across the interface, whereas the neighboring I27 (H1) forms contacts with L182 (H5′), Q148 (H4′), and I144 (H4′), and all these residues give rise to sharp or moderately broadened cross-peaks (e.g., Q148) at higher temperatures (Fig. 5). In addition, the T62 (H2)–C152 (H4) pair of interacting residues located one turn away from the cytoplasmic ends of the respective helices gives rise to sharp peaks at all temperatures (Fig. 5B). Last, the side chain of Y186 in the structured linker connecting helices H5′ and HE′ fits into the groove of the G30-XXX-G34 glycine zipper motif in H1 (*70*); its cross-peak remains sharp up to 60°C but undergoes a minor shift (~0.3 parts per million) and broadening at 62°C (Fig. 5B), likely influenced by the destabilization of the loop C β-turn–H5 interaction. Together, our data indicate a largely intact tetramer and a monomer helical bundle of the intermediate even after unfolding of loop C, owing to the large surface area at the interface, which contributes to the protein's stability through multiple van der Waals interactions.

## DISCUSSION

Studying membrane protein (MP) thermodynamic stability is challenging, as achieving a true equilibrium between the folded and unfolded states is often difficult, especially under the conditions of a lipid bilayer. Studying the kinetic stability of MPs may be even harder, as most of them are extremely stable under physiological conditions. Recently, we developed an approach of dissecting structural stabilization factors of helical MPs by combining HDX at predenaturation temperatures, where unfolding is largely reversible, and ssNMR. This approach allowed us to follow the progression of thermal unfolding of a seven-helical membrane protein *Anabaena* sensory rhodopsin at the atomic resolution and to identify its heat-resistant core (*71*).

Here, we presented further advancement of this methodology that allows following temperature-dependent kinetics of unfolding of a membrane protein at the level of individual amino acid residues. Through site-specific measurement of HDX rates, we identified the unfolding domains in hAQP1 that exchange in an internally correlated manner, thereby providing experimental evidence for employing the EX1 regime in our analysis of the HDX rates and for estimating activation energies of cooperative unfolding steps involving specific protein domains.

Our data point at a special role of the extracellular loop C, which forms the first unfolding domain through a network of interactions with the neighboring helices. Its melting precedes the unfolding of the TM core. Loop C is stabilized by an internal β-turn and maintains important hydrogen-bonding interactions with the extracellular ends of several helices, which are conserved within the aquaporin family across many taxa. We found that the cooperative unfolding of this extracellular domain has a high activation barrier (at least 300 kJ/mol) and is likely to involve not only the breakage of several very strong hydrogen bonds (such as those between the side chains of N127 and S196 and A130-V133, among others) but also a solvent exposure of several hydrophobic side chains (such as Val and Leu) of loop C and the adjacent helical ends. Once this extracellular domain is unfolded, an intermediate state with native-like TM interhelical packing is formed, from which the rest of the TM bundle can be cooperatively melted but with higher activation barrier (at least 600 kJ/mol), consistent with its larger size and a higher number of intradomain interactions.

The activation energies *E*_a_ we report here are the temperature-independent barriers that need to be overcome for the overall unfolding reaction (Fig. 3A). Their high values agree with the kinetic parameters derived from other measurements of thermal stability of α-helical membrane proteins. For example, the calorimetric measurement of the purple membrane in which bacteriorhodopsin trimers are arranged in a hexagonal 2D lattice shows that thermal denaturation of bacteriorhodopsin has an enthalpy of 100 to 179 kcal/mol (418 to 749 kJ/mol) (*72*); activation energies of unfolding estimated from the thermal inactivation functional assays were found to be 123 to 159 kcal/mol (514 to 665 kJ/mol) for the Na^+^,K^+^–adenosine triphosphatase (ATPase) depending on the type of a ligand (*73*), ~222 kJ/mol for the Ca^2+^ ATPase (*74*), ~231 kJ/mol for the Cu^+^ ATPase CopA (*75*), and ~320 kJ/mol for the photosynthetic reaction center (*76*).

The existence of the two consecutive high activation barriers provides a higher degree of kinetic stability to hAQP1, where the first native-like intermediate may serve as a “buffer” state. Abolishing the interactions involving N127 and V133 by mutagenesis (the N127A and V133P mutants) results in marked changes in the protein secondary structure and decreases its stability. The special role of loop C and its interactions resonate well with the three-stage MP folding model, in which the first two stages of helical insertion and assembly are followed by the third stage involving additional stabilization by ligands and loops (*13*). Both N127-S196 and A130-V133 interactions appear to be critical for maintaining proper hAQP1 fold as supported by our mutagenesis studies.

In the context of the HDX theory originally formulated by Linderstrøm-Lang (*48*), the protein undergoes a dynamic transition between the closed and the exchange-competent states. In our two-step unfolding model, there are two exchange-competent states of hAQP1. The first state is represented by the unfolding intermediate with transiently unfolded and exchange-competent loop C, part of helix HE and the cytoplasmic ends of H5 and H2, but with the TM domain in the native-like exchange-resistant conformation with a limited level of hydration. Here, we could draw some parallels with globular proteins. Their folding is driven by hydrophobic effects and was proposed to proceed through partially folded intermediate wet and dry molten globule states, characterized by high and low levels of hydration, respectively (*77*). In this sense, the unfolding intermediate of hAQP1 with exchange-resistant TM domain is akin to the dry molten globule state observed for globular proteins.

The second exchange-competent state exposes TM helices and may correspond to simultaneous breakage of both intramonomer, intermonomer, and intertetramer interhelical contacts within small 2D crystalline domains previously detected in our samples (*35*). Such a scenario would be consistent with the observed symmetry of the exchange: The TM residues located on all sides of helices, e.g., those facing the interior of the monomer, the intermonomer interface, and the periphery of the tetramer, exchange at similar rates.

## MATERIALS AND METHODS

### Experimental design

The experimental design is described in Fig. 1 and the associated text (see above). In brief, hAQP1 thermal unfolding was followed site-specifically by combining HDX and ssNMR. It was assumed that the unfolding results in an increased exchangeability of individual backbone and sidechain protons, detected via ssNMR. Both the temperature and time of incubation in D_2_O were varied to gain the information on kinetics, energetics, and structure of folding subdomains.

### Sample preparation

Uniformly ^15^N,^13^C-labeled WT hAQP1 (UCN WT-hAQP1) was produced as described previously (*36*). In brief, the expression vector pPICZB-hAQP1-Myc-His6 encoding full-length hAQP1 with a C-terminal Myc and His_6_-tags was transformed into the protease-deficient *Pichia pastoris* strain SMD1168H (Invitrogen) by electroporation. *P. pastoris* cells transformed with this vector were replated on yeast extract-peptone-dextrose plates for screening. A single well-isolated colony was first transferred to ^13^C,^15^N isotope–labeled buffered minimal dextrose media for small-scale growth before the methanol induction and large-scale growth in ^13^C,^15^N isotope–labeled buffered minimal media. Cells were broken via glass bead vortexing, solubilized in *n*-octyl-β-d-glucopyranoside detergent, and purified using Ni^2+^-NTA resin. Final reconstitution into proteoliposomes containing L-α-phosphatidylcholine (PC)/L-α-phosphatidylserine (PS) lipids [egg PC/brain PS = 9:1 (w/w); Avanti Polar Lipids] was completed via dialysis with a protein/lipid weight ratio of 2:1 (molar ratio of ∼1:20). UCN WT-hAQP1 proteoliposomes estimated to contain ~2 mg of protein were center packed in a 1.9-mm rotor (Bruker Biospin) for NMR experiments. The WT-hAQP1 samples for DSC measurements were prepared separately using the same media with natural isotopic abundance (NA).

Preparation of N127A and V133P mutants followed the same protocol described above except for the following: *n*-dodecyl-β-d-maltopyranoside was used for solubilization, and the detergent removal was achieved by adding bio-beads to the reconstituted protein suspension. To ensure consistency, the WT-hAQP1 for the FTIR measurements was prepared the same way as the mutants. Additional details are provided in the Supplementary Materials.

### DSC experiments

DSC experiments were performed on a NA WT-hAQP1 proteoliposome suspension in the D_2_O-based exchange buffer (pD 7) using a TA Nano DSC 602000.901 unit. The sample cells were pressurized to 3 atm, and the scan temperature range was 20° to 80°C at the heating rate of 1°C per min. The pH-dependence experiments were carried out at pHs of 6.0, 7.0, and 8.0 in H_2_O-based buffer (25 mM bis-tris propane and 10 mM NaCl, pH adjusted at 25°C). Additional details are provided in Supplementary Materials.

### NMR experiments and data analysis

Thermal unfolding of hAQP1 and H/D exchange were achieved by incubating an uncapped NMR rotor containing hAQP1 sample in a Thermowell PCR tube filled with a D_2_O-based buffer (pD 7) at controlled temperatures. The exchange process was quenched by transferring the rotor to ice and withdrawing D_2_O at the end of incubation. Two types of H/D exchange experiments were performed. For the exchange rate measurements, four separately prepared samples were incubated at 56°, 57°, 58°, and 59°C at increasing times. For temperature-dependent progression, the sample was subjected to D_2_O at 20°C for 16 hours and for 2 min at 55°, 60°, 62°, and 64°C. Temperature and time were controlled via a programmed Eppendorf Mastercycler Personal unit. Additional details are given in the Supplementary Materials.

All NMR experiments were carried out on a Bruker Avance III 800-MHz spectrometer using a Bruker 1.9-mm MAS triple-resonance ^1^H/^13^C/^15^N probe. Spectra were recorded at an MAS rate of 40 kHz and at a sample temperature of ~5°C. 2D NCA and NCO correlation spectra were recorded using Afterglow method (*78*) (fig. S10A) following each incubation time point. A short ^1^H/^15^N CP (*45*) time of 300 μs was used for the initial excitation to ensure that polarization transfer is mainly from the directly bonded amide proton to nitrogen. 2D ^13^C-^13^C correlation spectra were recorded using dipolar recoupling enhanced by amplitude modulation (DREAM) scheme (fig. S10B) (*79*). Swept low power two pulse phase modulation (slpTPPM) (*80*) proton decoupling was used during direct and indirect signal acquisition.

Carbon and nitrogen chemical shifts were indirectly referenced to 2,2-dimethyl-2-silapentane-5-sulfonic acid (*81*). All spectra were processed in Bruker TopSpin 3.6.1. Noise calculations and peak amplitude extractions were performed using CARA software (*82*). Chemical shift assignments of hAQP1 reported in the previous study (BMRB 26805) (*35*) were used in the data analysis. The structure analysis and visualization were performed using UCSF Chimera (*69*).

All the data fittings were performed using OriginLab software. Exchange rates of backbone amides were extracted by fitting cross-peak amplitudes to a single exponential decay as a function of the incubation time. The decay rates of NH_2_ groups were fitted using a decay function outlined in eq. S3. The temperature-dependent backbone amide H/D exchange rates were fitted to the Arrhenius equation (eq. S4) and the activation energies of unfolding were determined from the fitting parameters.

### Temperature-dependent ATR-FTIR experiments

FTIR measurements on hAQP1 in proteoliposomes were conducted on a temperature-controlled germanium attenuated total reflectance (ATR) accessory installed in a Vertex 70 FTIR spectrometer. The probed temperatures for FTIR measurements were 25°, 40°, 50°, 55°, 60°, and 65°C.

For all samples (WT-hAQP1, N127A, and V133P), a control spectrum was recorded at 25°C after drying the proteoliposomes resuspended in H_2_O-based exchange buffer on the surface of the germanium crystal. Temperature-dependent H/D exchange was accomplished by rehydrating the sample with pure D_2_O and raising the temperature to the target point at which the sample was incubated for 2 min. The sample was then subsequently cooled down back to 25°C and dried again before each spectrum was collected. For the WT-hAQP1 sample, an additional set of temperature-dependent spectra was recorded in H_2_O to monitor the secondary structure changes. Additional details and references are given in the Supplementary Materials.

### Statistical analysis

Adjusted *R*^2^ regression was used to estimate errors. Standard fitting errors and spectral noise are addressed in the individual figure legends.
