# Supplementary material for: Probing the energy barriers and stages of membrane protein unfolding using solid-state NMR spectroscopy
Source: Sci Adv. 2024 May 17;10(20):eadm7907. doi: 10.1126/sciadv.adm7907 (PMC11639674; doi:10.1126/sciadv.adm7907)
Supplement: Supplementary file 1 — Supplementary Text Figs. S1 to S10 Tables S1 to S3 [file sciadv.adm7907_sm.pdf]

Science Advances
AAAS

Supplementary Materials for

Probing the energy barriers and stages of membrane protein unfolding using solid-state NMR spectroscopy

Peng Xiao *et al.*

Corresponding author: Leonid S. Brown, lebrown@uoguelph.ca; Vladimir Ladizhansky, vladizha@uoguelph.ca

Sci. Adv. **10**, eadm7907 (2024)
DOI: 10.1126/sciadv.adm7907

This PDF file includes:

Supplementary Text
Figs. S1 to S10
Tables S1 to S3

Supplementary Text

Material and Methods

Common chemicals of a reagent grade for protein expression, isolation, and reconstitution were purchased from either Fisher Scientific (Unionville, Ontario, Canada) or Sigma-Aldrich (Oakville, Ontario, Canada). ^{15}N labeled ammonium sulphate, $^{13}C_6$-labeled glucose, and ^{13}C-labeled methanol were purchased from Cambridge Isotope Laboratories (Andover, MA, USA). The Ni^{2+}-NTA (nitrilotriacetic acid) agarose resin was purchased from Qiagen (Mississauga, Ontario, Canada). Egg phosphatidylcholine (PC) and brain phosphatidylserine (PS) were purchased from Avanti Polar Lipids (Alabaster, AL) as chloroform solutions (>99% purity) and used without further purification. The lipid compositions and their phase transition temperatures can be found at:
https://avantilipids.com/product/840032
https://avantilipids.com/product/840051
https://avantilipids.com/tech-support/physical-properties/phase-transition-temps

Sample preparation

Uniformly ^{15}N, ^{13}C-labeled wild-type hAQP1 (UCN WT-hAQP1) was prepared as described previously (*36*). Briefly, the expression vector pPICZB-hAQP1-Myc-His6 encoding full-length hAQP1 with a C-terminal Myc and His_6-tags was transformed into the protease-deficient *Pichia pastoris* strain SMD1168H (Invitrogen) by electroporation.

Cell stocks stored at -80 °C following the original transformation were replated onto yeast extract−peptone−dextrose (YPD, 1% yeast extract, 2% peptone, 2% dextrose, pH 6.5) agar plates with high concentration of zeocin (Cedarlane, 1000 μg/mL) and incubated at 30 °C. After 4–7 days of growth, a single well-isolated colony was transferred to 50 mL ^{13}C,^{15}N isotope-labeled buffered minimal dextrose media (BMD, 1% $(^{15}NH_4)_2SO_4$, 0.5% $^{13}C_6$-labeled dextrose, 0.00004 % biotin, 0.34 % yeast nitrogen base, potassium phosphate 100 mM, pH 6.0) in a 250 mL sterile shake-flask and grown at 30 °C for 18–24 hours. Cultures were then transferred to 1 L sterile shake-flasks containing additional 200 mL BMD media and grown at 29 °C and 275 rpm for another 18–24 hours. Cells were collected by centrifugation at 1500×g rcf for 10 minutes at 4 °C in sterile containers and resuspended in 1 L ^{13}C,^{15}N isotope-labeled buffered minimal methanol media (BMM, same ingredients as BMD except dextrose was replaced with ^{13}C-methanol, pH 6.0) in a sterile 2.8 L shake-flask. Cells were grown for additional 24 hours at 28 °C, 240 rpm, and then collected by centrifugation at 1500×g rcf for 10 minutes at 4 °C and washed twice with MilliQ water. Cell pellets were stored at -20 °C for later use.

Frozen cell pellets were resuspended in one pellet volume of cell resuspension buffer (CRB, 20 mM Tris-HCl, 100 mM NaCl, 0.5 mM EDTA, 5 % (w/v) glycerol, pH 7.6) with addition of equivalent volume of acid-washed glass beads (Fisher, 420–600 μm diameter). Cells were broken by vortex mixing for one minute followed by a one-minute interval in which the suspension was rested on ice; this pattern was repeated 4 times, following which cell debris was separated via low-speed centrifugation at 700×g rcf for 5 minutes at 4 °C and cell lysate was collected. Eight

rounds of vortexing were completed with collection of additional lysate for complete breakage. Combined supernatants containing cell lysate from all rounds of breakage were centrifuged at 150,000×g rcf for 1 hour at 4 °C, and the pellet containing membrane fractions was stored at -20 °C for later use.

Solubilization of hAQP1 was achieved by resuspending the frozen membrane fraction pellet in 20 mL solubilization buffer (20 mM Tris-HCl, 100 mM NaCl, 20 % (w/v) glycerol, 10 mM imidazole, Sigma protease inhibitor cocktail tablet 1X ~ 40 mL of buffer, pH 8.0) with 5 % n-octyl-β-D-glucopyranoside (OG) detergent and stirring gently at 4 °C for 2 hours. Unsolubilized material was removed by centrifugation at 150,000×g rcf for 1 hour, and 2–5 mL Ni^{2+}-NTA resin (Qiagen) was then added to the supernatant containing solubilized hAQP1. This solution was incubated overnight with gentle stirring to allow complete resin binding, then placed in a column and the buffer was flown through. The resin was washed with 500 mL washing buffer (same ingredients as solubilization buffer except the imidazole concentration increased to 30 mM, pH 8.0) with 1 % OG detergent and hAQP1 was eluted using 20–40 mL elution buffers (same as washing buffer except for different imidazole concentrations, pH 8.0) with increasing concentrations of imidazole (150–500 mM). Purified hAQP1 in elution buffer was stored at 4 °C for no more than 7 days, during which the elution volume was concentrated to 5 mL with concurrent removal of imidazole (Amicon centrifugal concentrator, 10 kDa cut-off, Fisher) and buffer exchange to reconstitution buffer (100 mM NaCl, 50 mM potassium phosphate, pH 7.5).

For lipid reconstitution, purified hAQP1 in imidazole-free reconstitution buffer was mixed with a lipid stock (egg PC:brain PS, 9:1 w/w, Avanti lipids) at an approximate protein to lipid weight ratio of 2:1 (molar ratio of~1:20), and incubated overnight with gentle stirring at 4 °C. For detergent removal, the sample was placed into a dialysis bag (12–14 kDa cutoff, Spectra/Por, VWR) fixed at the top of a container with 200-500 mL dialysis buffer (300 mM NaCl, 50 mM potassium phosphate, pH 7.5) and 8 g of Bio-beads SM-2 (Biorad), and the dialysis container was stirred gently at 4 °C. Dialysis continued for 7-10 days with replacement of the dialysis buffer and addition of 1–4 g Bio-beads every 24 to 48 hours, until no detergent was present. The reconstituted proteoliposome suspension was collected from the dialysis bag by centrifuging at 150,000×g rcf for 1 hour at 4 °C, followed by buffer exchange with NMR buffer (25 mM Tris-HCl, 10 mM NaCl, pH 7.0) and an additional 300,000×g rcf centrifugation for 30 minutes at 4 °C. Finally, proteoliposomes in NMR buffer were centrifuged at 900,000×g rcf for 16 hours at 4 °C and the excess buffer was withdrawn from the pellet. The resulting pellet was stored at -20 °C until further use.

The preparation for the mutant constructs (N127A and V133P) followed the protocol for the WT described above with minor modifications to optimize the yield as outlined below. The mutants were transformed using the same transformation protocol as noted above, the major differences only occurring during the solubilization and purification steps: 2.5% concentration of n-dodecyl-β-D-maltopyranoside (DDM) was used during solubilization instead of OG; 0.05% DDM was used for both the wash buffer and elution buffer; no protease inhibitor was added in the wash buffer but 1 Roche protease tablet/30 mL of buffer was used for the elution buffer; 0.02% DDM was used in the reconstitution buffer. The detergent removal was achieved by adding Bio-beads to the reconstituted protein suspension gently nutated on an Orbitron shaker at 4 °C for a total of 48 hrs. During the first 24 hrs, the Bio-beads were added at a concentration of 0.8 g/mL in three

equal but separate additions; these additions were performed over the course of ~9 hours, with one addition every 3 hours. After the first 24 hrs, an additional 0.2 g/mL of Bio-beads was added to the proteoliposome suspension to facilitate the complete removal of detergent. After the full 48 hrs of incubation on Orbitron at 4 °C, the proteoliposomes were separated from the Bio-beads using 18- and 22- gauge needles, and collected via centrifugation at 300,000xg, for 30 mins. The resulting proteoliposome pellets were stored at -20 °C prior to FTIR measurements. To ensure consistency, the WT for the FTIR measurements was prepared the same way as the mutants.

UCN WT-hAQP1 proteoliposomes estimated to contain ~2 mg protein in NMR buffer were center-packed in a 1.9 mm rotor (Bruker Biospin) for NMR experiments. The samples used for DSC measurements were prepared separately using the same procedures as for the NMR sample described above except that the media was at natural isotopic abundance (NA). For the FTIR measurements, NA WT-hAQP1, NA N127A and NA V133P mutants were prepared using the mutant protocol described above.

Thermal denaturation and H/D exchange experiments

Thermal unfolding of hAQP1 and H/D exchange was achieved by incubating the sample in a D_2O based buffer at controlled temperatures. For each incubation, the NMR rotor packed with UCN hAQP1 sample was uncapped and placed in a 0.5 mL Thermowell PCR tube filled with the D_2O based exchange buffer (10 mM NaCl, 25 mM Bis-tris propane, pD 7). The PCR tube was then transferred to the heating block of an Eppendorf Mastercycler Personal unit preheated and equilibrated at the target temperature. After the set incubation time (see below), the PCR tube was quickly removed from the heating block and submerged in ice to prevent any additional thermal denaturation. The excess buffer within the NMR rotor was first syphoned out using the tip of Kimwipes tissues and further removed by placing the NMR rotor under high vacuum in a desiccator for 10–15 minutes. The NMR rotor was then re-sealed with the cap before transferring back to the NMR probe. The sufficient hydration levels were verified by proton NMR detection of residual water and preserved narrow linewidth of carbons, which are sensitive to desiccation. The additional exposure to residual D_2O for 10–15 minutes at room temperature results in negligible additional exchange effects for slow-exchanging residues as was confirmed by a direct comparison of the NCA and NCO spectra collected on a test sample before and after this exposure.

For the HDX rate measurements, 4 sets of experiments were carried out on 4 separately prepared UCN hAQP1 samples at the incubation temperatures of 56 °C, 57 °C, 58 °C, and 59 °C. NMR spectra were collected after each incubation. Incubation time points for each temperature were as follows:

at 56 °C: 60s, 180s, 540s, 1620s, 4860s, 10980s, 28800s, 57600s, 86400s, and 172800s;
at 57 °C: 30s, 180s, 360s, 1080s, 3600s, 10800s, 32400s, and 57600s;
at 58 °C: 120s, 240s, 480s, 960s, 1960s, 3840s, 7680s, 15360s, and 30720s;
at 59 °C: 60s, 240s, 360s, 720s, 1440s, 2880s, 5760s and 8640s.

For the temperature-dependent series of ssHDX-NMR experiments, all experiments were carried out on the same UCN hAQP1 sample. The sample was incubated at elevated temperatures of 20 °C, 55 °C, 60 °C, 62 °C, and 64 °C, and subsequently cooled down to 5 °C for NMR detection after each incubation. The incubation time was 16 hrs at 20 °C and was reduced to 2 min for higher temperatures of 55 °C, 60 °C, 62 °C, and 64 °C.

The time for the sample to reach the target set temperature and equilibrate inside the heating block was estimated to be 15–30 s by directly placing the thermocouple into the PCR tube filled with buffer. This uncertainty limits the time resolution of our measurements for the fast-exchanging residues. However, its effect is negligible for residues exchanging on the time scales of thousands of seconds.

NMR experiments

All NMR experiments were carried out on a Bruker Avance III 800 MHz spectrometer using a Bruker 1.9 mm MAS triple resonance $^{1}H/^{13}C/^{15}N$ probe. All spectra were recorded at a MAS rate of 40 kHz and at a sample temperature of ~5 °C. Swept low power Two Pulse Phase Modulation (slpTPPM) (*80*) proton decoupling was used during direct and indirect signal acquisition. The experimental pulse sequences are shown in **Fig. S10**.

For the HDX rate measurements, a set of 2D ^{15}N-$^{13}C\alpha$ and ^{15}N-$^{13}C'$ (NCA and NCO) correlation spectra were recorded using Afterglow method (*78*) **(Fig. S10A)** following each incubation point. A short $^{1}H/^{15}N$ cross polarization (CP) (*45*) time of 300 μs was used for the initial excitation in all ^{15}N-^{13}C correlation experiments to ensure the polarization transfer is mainly between the directly bonded amide proton and nitrogen. A 3 ms dephasing period was used between the NCA and NCO segments to remove the undesired residual ^{13}C signal.

For the temperature-dependent series, a set of 2D NCA, NCO and 2D ^{13}C-^{13}C correlation spectra were recorded following each incubation experiment. A short 300 μs $^{1}H/^{15}N$ CP time was used for the initial excitation in both NCA and NCO experiments. The 2D ^{13}C-^{13}C correlation spectra were recorded with 2 ms $^{1}H/^{13}C$ CP excitation and 7 ms mixing using the DREAM scheme with a tangential mixing (*79*) (**Fig. S10B**) targeting the aliphatic carbons (**Fig. S8**). The DREAM mixing mostly resulted in peaks from one bond transfer (negative peaks); in some cases, peaks from two-bond transfers (positive peaks) and even three-bond transfers (negative peaks) could be observed.

Data processing and analysis

Carbon and nitrogen chemical shifts were indirectly referenced to DSS (2,2-Dimethyl-2-silapentane-5-sulfonic acid) by adjusting the shift of ^{13}C adamantane downfield peak to 40.48 ppm (*81*). All spectra were processed with Bruker Topspin using Lorentzian-to-Gaussian apodization functions. Noise calculations and peak amplitude extractions were performed using CARA software (*82*). The chemical shift assignments of hAQP1 reported in the previous study (BMRB: 26805) (*35*) were used in the data analysis.

All the data fittings were performed using the OriginLab software. For the backbone amides (N-H group), the amplitudes of cross peaks were fit to a single exponential decay as a function of the incubation time:

$$Y(t) = Y_0 + Ae^{-k_{HDX}t} \quad \text{(S1)}$$

Where the residual signal Y_0, the exchange rate k_{HDX} and the amplitude scaling factor A are the fit parameters.

For the side chain amide (NH_2 group) which possesses two directly bonded hydrogens, the possible states during the exchange (NH_2, NHD, NDH and ND_2) and their populations follow the expressions:

$$NH_2\text{:}\ \ e^{-k_1 t} \cdot e^{-k_2 t} \quad \text{(S2.a)}$$

$$NHD + NDH\text{:}\ e^{-k_1 t} \cdot (1 - e^{-k_2 t}) + (1 - e^{-k_1 t}) \cdot e^{-k_2 t} \quad \text{(S2.b)}$$

$$ND_2\text{:}\ (1 - e^{-k_1 t}) \cdot (1 - e^{-k_2 t}) \quad \text{(S2.c)}$$

Here, k_1 and k_2 are the exchange rates for each site. We assumed that protons exchange at the same rate ($k_1 = k_2 = k_{HDX}$), only NH_2 and HND configurations contribute to the CP signal and their $^1H/^{15}N$ CP efficiency are the same, leading to the decay function for the side chain amide exchange:

$$Y(t) = Y_0 + A(2e^{-k_{HDX}t} - e^{-2k_{HDX}t}) \quad \text{(S3)}$$

The data fitting was done with Levenberg-Marquardt iteration algorithm, and the standard errors of the fitting parameters were calculated following the OriginLab Error Propagation formula (see https://www.originlab.com/doc/en/Origin-Help/NLFit-Theory) with spectral noise as the Instrumental Weight in the weighting method.

Within the EX1 limit approximation, the activation energies (E_a) of unfolding were extracted by fitting the experimentally determined backbone amide hydrogen exchange rates at 4 different temperatures to the natural logarithm of the Arrhenius equation:

$$\ln(k_{HDX}) = \left(-\frac{E_a}{R}\right)\left(\frac{1}{T}\right) + \ln(A) \quad \text{(S4)}$$

Where R is the molar gas constant ($8.314\ J \cdot K^{-1} \cdot mol^{-1}$). No weighting was used in the linear regression and error propagation.

DSC Experiments

The DSC experiments were performed using a TA Nano DSC 602000.901 unit. Approximately 1 mg of NA WT-hAQP1 proteoliposomes with a protein to lipid ratio of 2:1 (w/w) was

resuspended in the D_2O-based exchange buffer (25 mM Bis-Tris propane, 10 mM NaCl, pD 7 adjusted at 25 °C) before loading into the DSC sample cell. The sample cell was pressurized to 3 atm, and the scanned temperature range was 20–80 °C at the heating rate of 1 °C/min. The midpoint temperature of the unfolding transition T_m was found to be in the ~62–64 °C range, with a pre-transition onset at ~50 °C. A PC/PS lipid mixture used for the hAQP1 proteoliposome reconstitution was prepared and measured separately, with approximately 1 mg of liposome in a H_2O-based buffer (potassium phosphate 50 mM, NaCl 100 mM, pH 7.5 adjusted at 25 °C). The scanned temperature range was 20–75 °C, among which no prominent transition was observed.

The pH dependence experiments were carried out with the same DSC experimental setup as described above, apart from the temperature scan range being 10 °C–90 °C. Three batches of WT-hAQP1 proteoliposomes were resuspended in H_2O-based buffers at pH's of 6.0, 7.0, and 8.0 (adjusted at 25 °C), respectively. The resulting thermograms were normalized to 1. The pH dependent stability is observed as the T_m differs between the pH points: while the T_m is at ~62 °C for pH 7.0, the T_m for pH 6.0 and 8.0 was found at ~57 °C and ~65 °C, respectively.

Temperature-dependent ATR-FTIR experiments

FTIR measurements of hAQP1 in proteoliposomes were conducted on a temperature-controlled Germanium Attenuated Total Reflectance (ATR) accessory (Pike Technologies, Madison WI) installed in a Vertex 70 FTIR spectrometer (Bruker, Milton ON). One hundred spectra were averaged at 4 cm^{-1} resolution, using a DTGS detector and transmission spectra of the empty ATR cell as reference. The probed temperatures for the temperature-dependent FTIR measurements were 25 °C, 40 °C, 50 °C, 55 °C, 60 °C, and 65 °C.

For all samples (WT-hAQP1, N127A, and V133P), a control spectrum was recorded at 25 °C after drying 15 μL (100–150 μg of protein) of the proteoliposomes resuspended in H_2O based exchange buffer (pH 7.0) on the surface of the germanium crystal under a gentle flow of dry nitrogen gas. The H/D exchange at room temperature was accomplished by aliquoting 2 μL of pure D_2O directly onto the previously dried sample and incubated for 10 mins on the crystal surface, before drying again with nitrogen gas. The spectrum corresponding to the H/D exchange at 25 °C was then recorded once the sample was fully dry. For each temperature point thereafter, another 2 μL of D_2O was aliquoted onto the crystal to rehydrate the sample before increasing the temperature to the desired point. Once the temperature equilibrated, the sample was incubated for 2 mins, then subsequently cooled down back to 25 °C and dried again with nitrogen gas. The corresponding spectrum was then recorded to observe any secondary structure changes in the Amide I band (~1655 cm^{-1}), and to follow the extent of the H/D exchange by monitoring the Amide II band (~1567 cm^{-1}) and the Amide II' band (~1445 cm^{-1}).

For the WT-hAQP1 sample, an additional set of temperature-dependent spectra was recorded without H/D exchange. All procedures are the same as described above except for pure D_2O being replaced by pure H_2O.

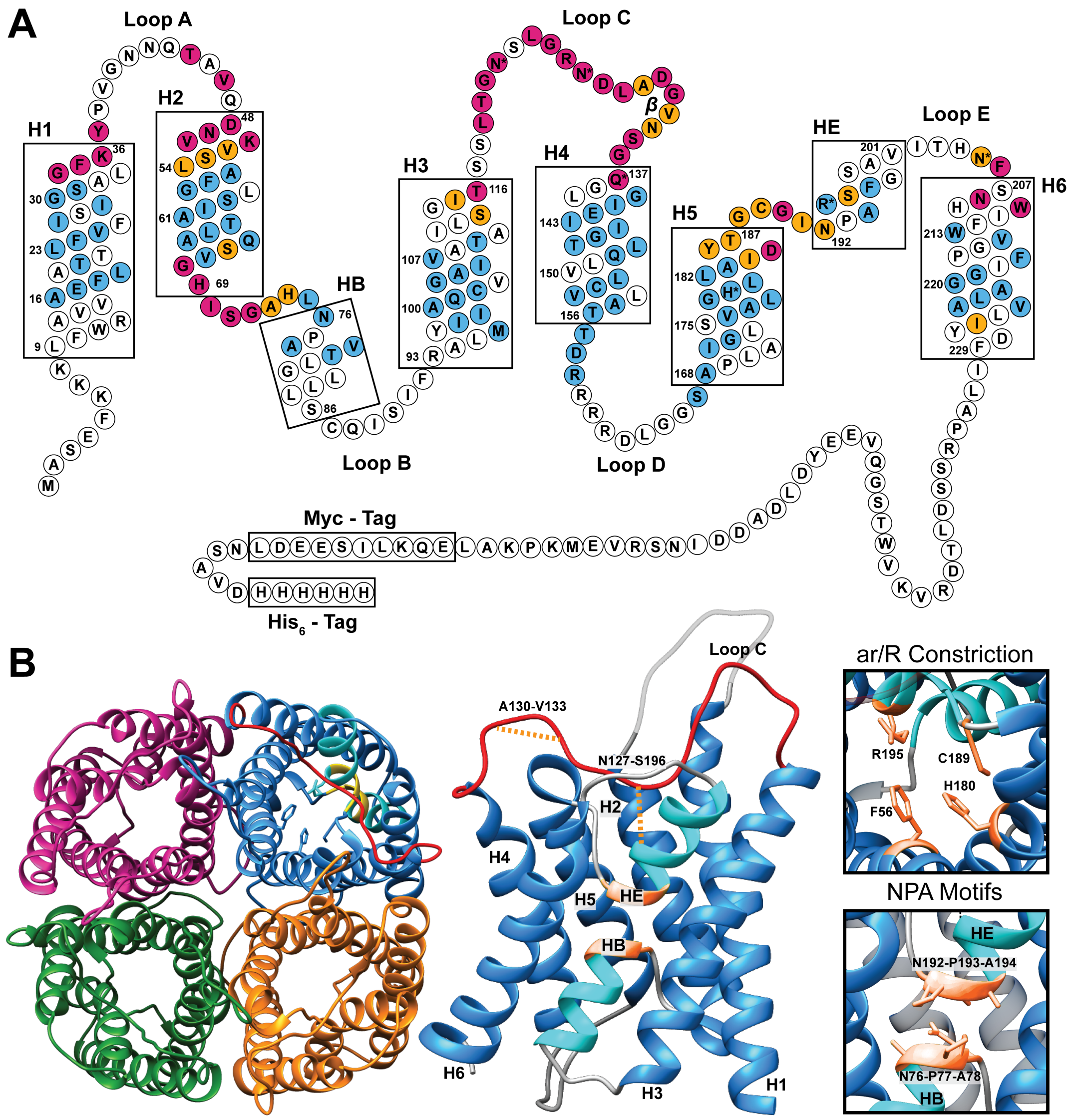

Fig. S1. Topology and 3D Structure model of hAQP1. (A) Topological model with helices, loops, the type II beta turn, the purification His$_6$-tag and Myc-tag labelled. The helical regions are indicated as rectangles. Backbone amides of 138 out of 192 assigned residues are resolved in the 2D NCA/NCO spectra and are coloured on the topological model. Residues with backbone peaks in the slow, intermediate and fast exchange time regimes are shown in blue, orange and magenta, respectively; residues with both backbone and side chain peaks resolved in the 2D spectra are marked with an asterisk sign. Empty circles indicate residues that are either not assigned or not resolved in the 2D spectra. (**B**) 3D models showing hAQP1 tetramer, monomer, the aromatic/arginine (Ar/R) constriction and the central NPA motifs. Each monomer is coloured differently. In the monomer model, loop C is highlighted in red, half-helices HE and HB are highlighted in cyan and the NPA motifs are highlighted in orange. Orange dashed lines indicate the A130(O)-V133(N) backbone and the N127-S196 sidechain-sidechain hydrogen bonds. The homology model of hAQP1 is based on the high-resolution structure of bovine AQP1 (PDB 1J4N) constructed using SWISS-MODEL (https://swissmodel.expasy.org).

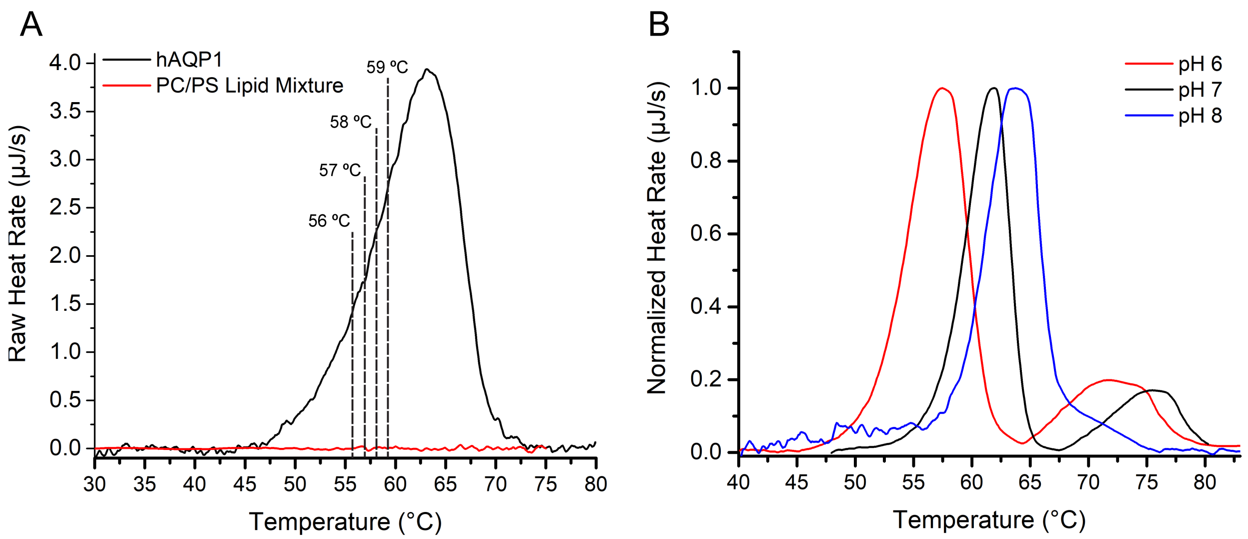

Fig. S2. Differential Scanning Calorimetry measurements of the WT-hAQP1. (**A**) DSC heating curves of WT-hAQP1 proteoliposomes in D_2O-based buffer at pD 7 (black) and the PC/PS lipid mixture in H_2O-based buffer at pH 7.5 (red). Dashed lines indicate the incubation temperatures at which exchange rates were probed. (**B**) DSC heating curves of WT-hAQP1 proteoliposomes in H_2O-based buffer at pH 6, 7, 8, respectively. The resulting thermograms were normalized to 1. All curves are presented after baseline correction.

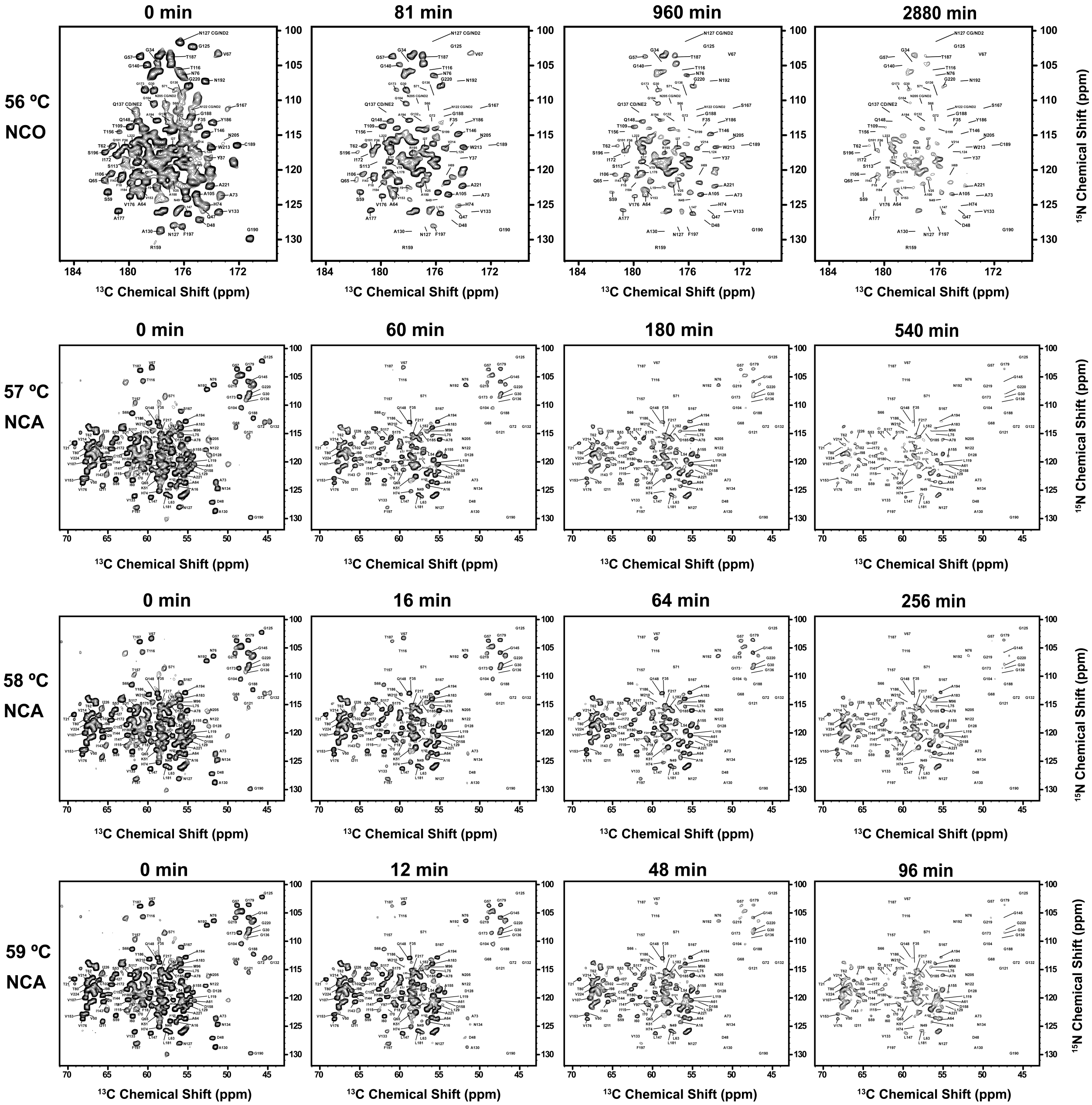

Fig. S3. Representative examples of the 2D NCA and NCO spectra following time-dependent H/D exchange progression. The first contour in all spectra is at 5 times root-mean-square of the noise and the contour multiplication factor is at 1.2.

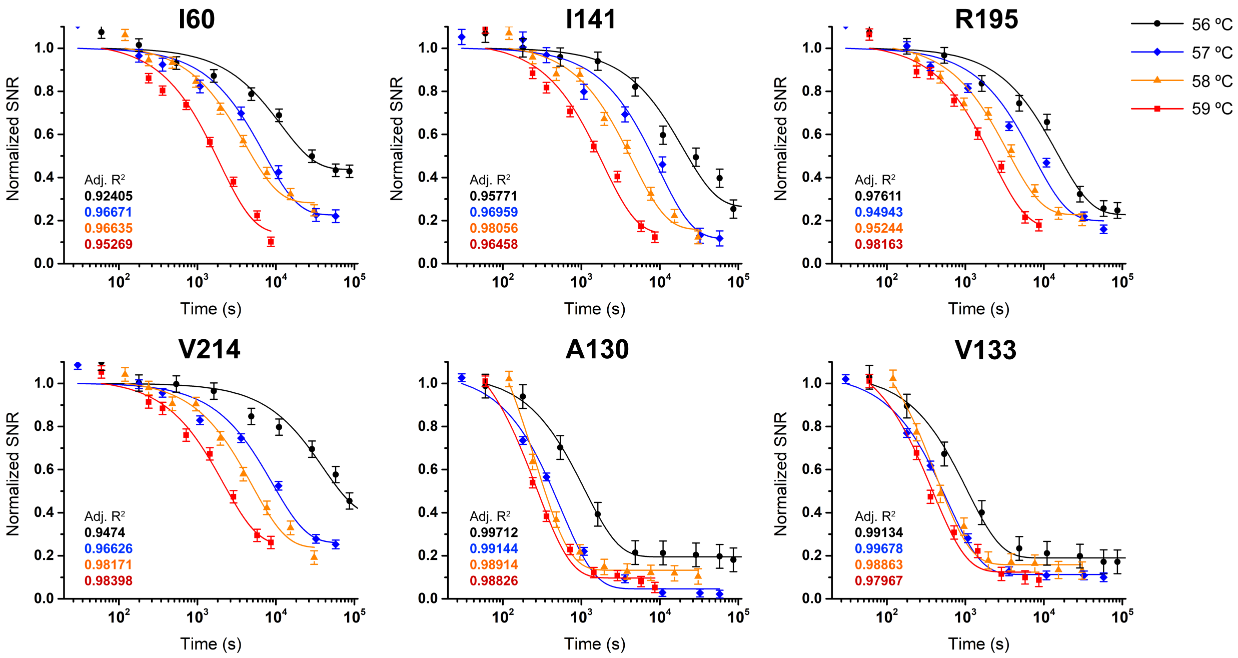

Fig. S4. Representative HDX signal decay of backbone amide and their best single exponential decay fits. The time axis is shown on the logarithmic scale. Errors are estimated from spectral noise.

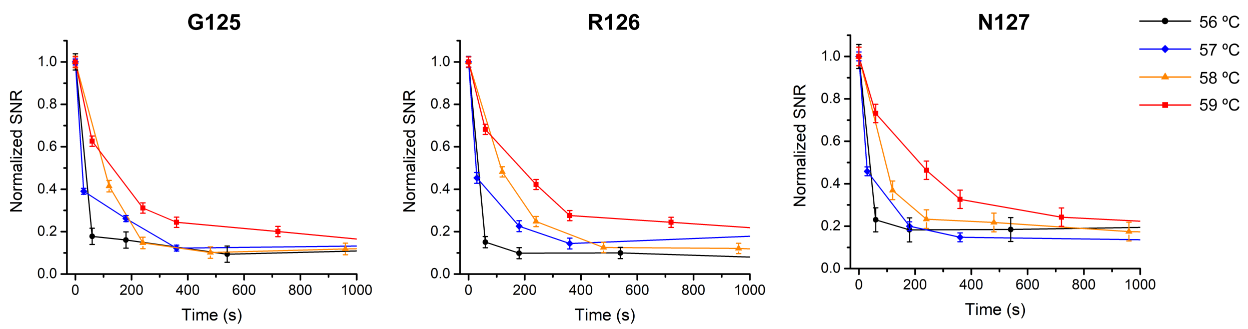

Fig. S5. Representative examples of HDX trajectories for backbone amides of loop residues. The signals from the backbone amides of these residues drop to noise level within a few minutes of incubation at temperatures of 56-58 ºC. Errors are estimated from spectral noise.

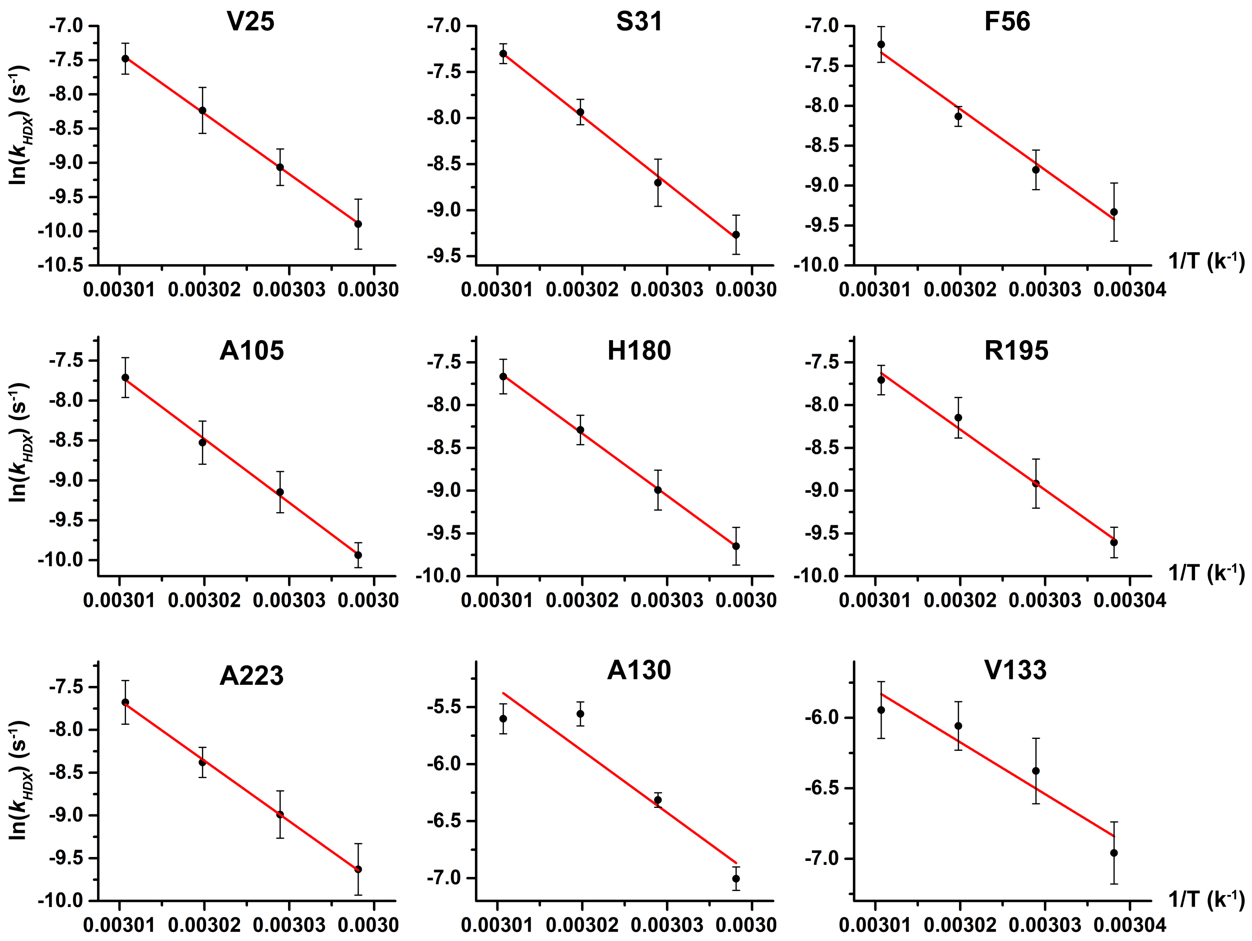

Fig. S6. Representative results for the activation energy fitting. The exchange rate data at four different temperatures were fit to the natural logarithm of the Arrhenius equation. Errors are one standard deviation.

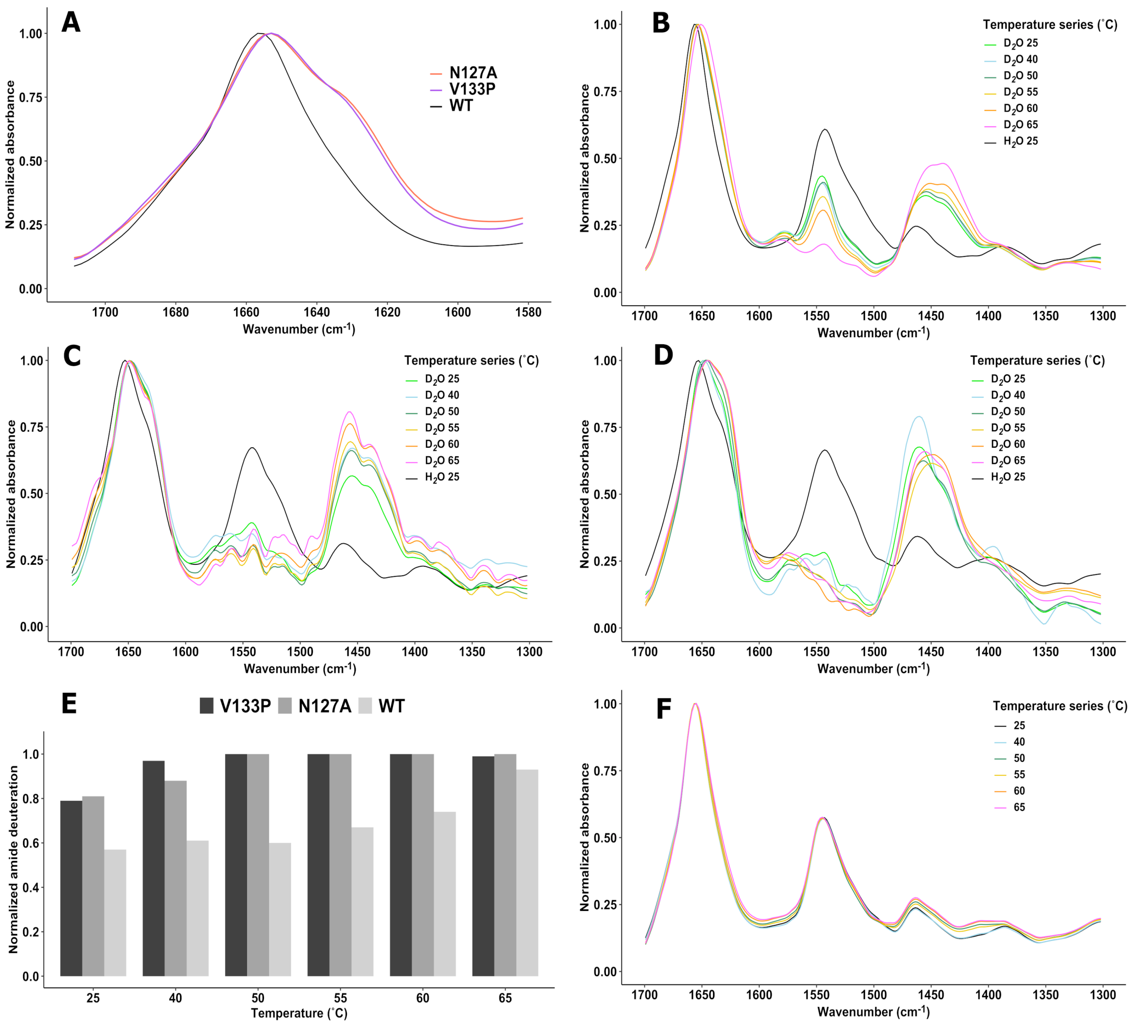

Fig. S7. FTIR measurements of HDX and secondary structure for the WT and mutants of hAQP1. (**A**) Amide I band comparison between the WT, N127A, and V133P mutants of hAQP1 in H_2O at room temperature. (**B-D**) Temperature-dependent spectra in D_2O for the WT (**B**), V133P (**C**), and N127A (**D**), showing the progression of HD exchange. (**E**) The Amide II integrated intensities showing the extent of deuteration for all the samples. The extent of deuteration was estimated as $\mathbf{1-\frac{\mathit{I}_{\mathit{Amide\ II,D_2O}}}{\mathit{I}_{\mathit{Amide\ II,H_2O}}}}$, where $\boldsymbol{I_{Amide\ II,D_2O}}$ and $\boldsymbol{I_{Amide\ II,H_2O}}$ are the integrated Amide II band intensities in the D_2O spectrum and in the reference H_2O spectrum, respectively (**F**). Temperature-dependent spectra of WT in H_2O. No shift was observed for both Amide I and II bands.

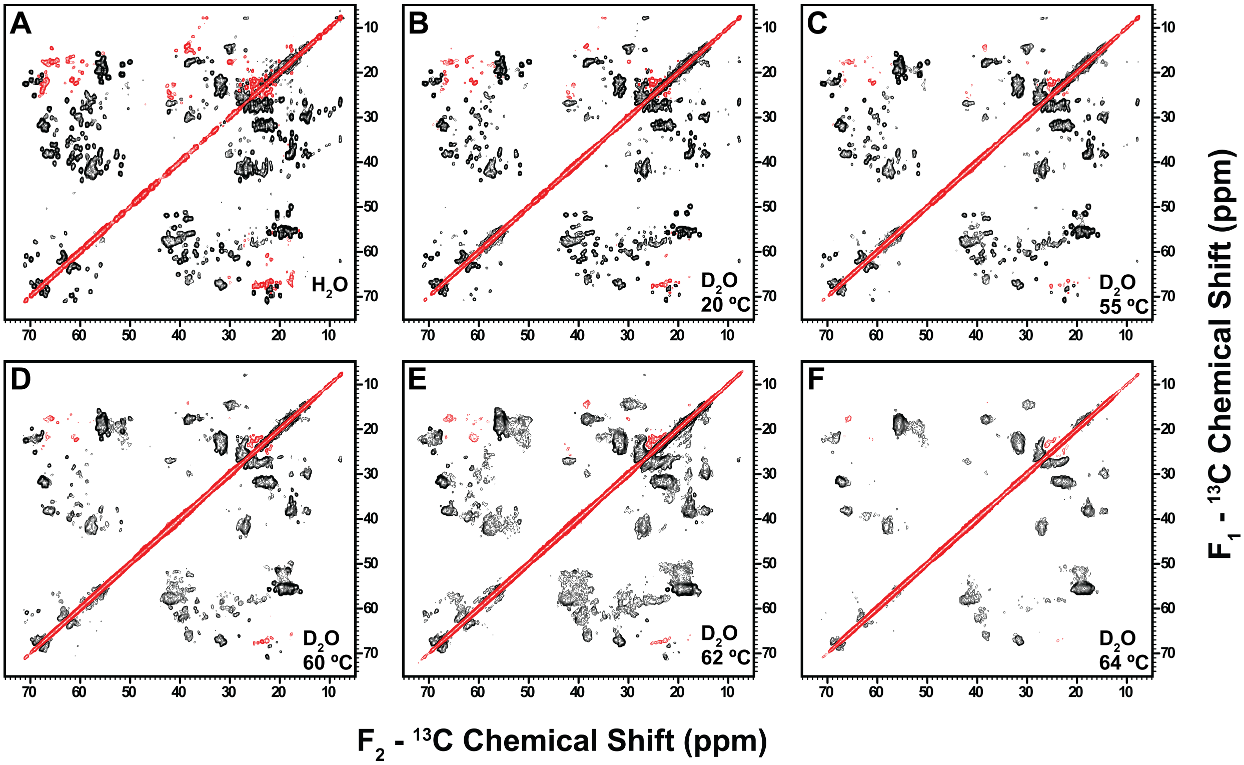

Fig. S8. A series of 2D ^{13}C-^{13}C DREAM correlation spectra of hAQP1 collected as a function of incubation temperature. The incubation conditions i.e., buffers and temperatures, are indicated in each panel. Cross-peaks from one- or three-bond transfers are in black; the diagonal and two-bond correlations are in red. The first contour is at 5 times root-mean-square of the noise in (**A**), and 4 times root-mean-square of the noise in (**B-F**). The contour multiplication factor is 1.2 in all spectra.

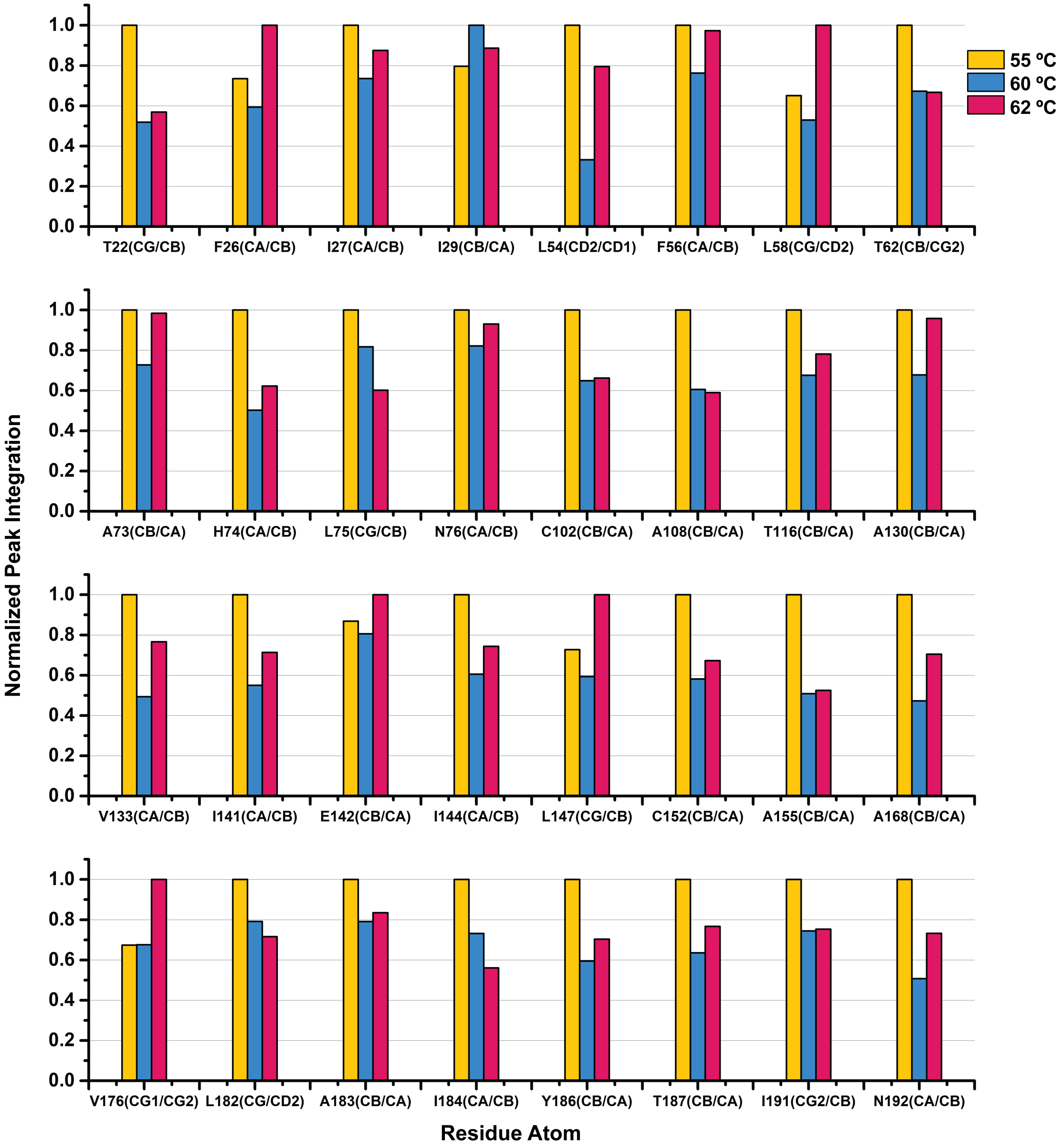

Fig. S9. Representative examples of carbon-carbon cross-peak integrated intensities. Integrated peak intensities are normalized with respect to the high value for each peak.

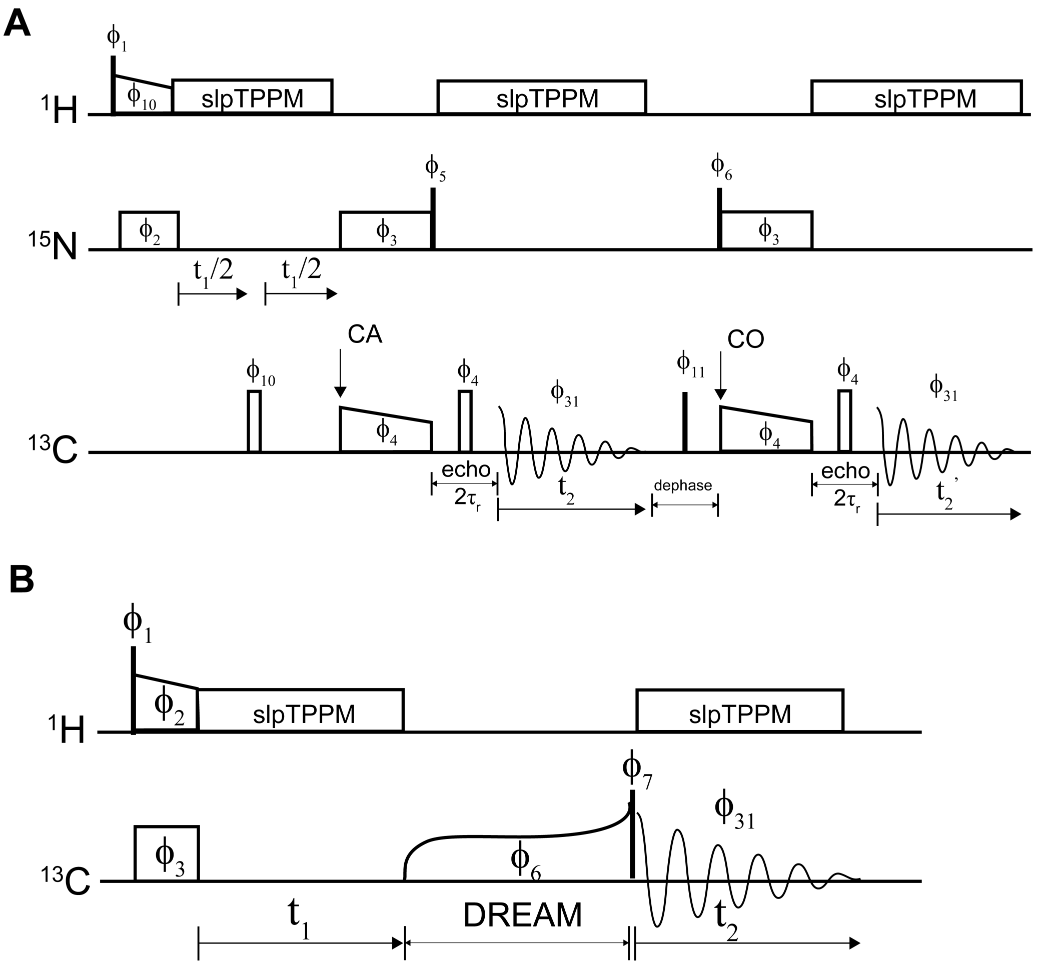

Fig. S10. NMR pulse sequences. (**A**) Sequence for 2D NCA and NCO experiments using "Afterglow" method. (**B**) Sequence for 2D ^{13}C -^{13}C correlation experiments using the DREAM mixing scheme. Solid and hollow bars represent $\pi/2$ and π pulses, respectively; τ_r is the rotor period. SlpTPPM decoupling is used during the chemical shift evolution of both direct and indirect dimensions. In (**A**), the initial $^1H/^{15}N$ CP time was 300 μs; the dephasing period between the NCA/NCO segments was set to 3 ms. In (**B**), the DREAM sweep is a tangential variation of the RF amplitude from 37.5% - 62.5% of ω_r over a period of 7 ms. The phase cycling tables are as follows: (**A**) φ_1 = (y, -y), φ_2 = (x), φ_3= (x), φ_4 = (x, x, y, y, -x, -x, -y, -y), φ_5 = (y), φ_6 = (-y), φ_{10} = (x), φ_{11} = (x), φ_{31} = (x, -x, y, -y, -x, x, -y, y); (**B**) φ_1 = (x, x, x, x, -x, -x, -x, -x), φ_2 = (y), φ_3 = (x), φ_6 = (x), φ_7 = (x, -x), φ_{31} = (x, x, x, x, -x, -x, -x, -x).

Table S1. Conserved motifs found in the AQP family.

Protein	Structure		Beta II turn	Beta - H5	HE - Loop C		H4 - H2/HB loop - H5	H1 - H5/HE loop - H3
hAQP1	6POJ	ssNMR	A130-V133	A130-T187	R195-G125		E142-G190/I191-T187	E17-H74/L75/S71-Q101
	1IH5	EM 3.70 Å	A130-V133	A130-T187		R195-N127		
	1H6I	EM 3.54 Å	A130-V133		R195-G125	S196-N127		
	4CSK	Xray 3.28 Å	A130-V133	A130-Y186		S196-N127		
bAQP1 (*Bos taurus*)	1J4N	Xray 2.20 Å	A132-V135	A132-Y188	R197-G127	S198-N129	E144-G192/I193-T189	E17-H76/L77/S73-Q103
hAQP2	4NEF	Xray 2.75 Å	S122-T125	S122-Y178	R187-A117	S188-N119	E134-S182/M183-T179	E16-H66/I67/S63-Q93
hAQP4	3GD8	Xray 1.80 Å	H151-L154	H151-Y207	R216-G146	S217-T148	E163-S211/M212-T208	E41-H95/I96/S92-Q122
hAQP5	3D9S	Xray 2.00 Å	N123-N125	N123-F179	R188-A118	S189-N120	S183/M184-E135-T180	E17-H67/I68/S64-Q94
Aqy1 (*Pichia pastoris*)	3ZOJ	Xray 0.88 Å	G163-L166	G163-Y218	R227-F158	S228-N160	E175-G222/L223-T219	E51-N110/L111/S107-Q137
AQPZ (*Escherichia coli*)	1RC2	Xray 2.50 Å	S125-G128	G127-P179	R189-A117	S190-N119	S184/V185-E138-T181	E8-H61/F62/S58-Q88
SoPIP2;1 (Spinach)	1Z98	Xray 2.10 Å	A156-Y159	A156-I216	R225-G151	S226-N153	E168-G220/I221-T217	E44-H99/I100/S96-Q126
AqpM (*Methanothermobacter marburgensis*)	2F2B	Xray 1.68 Å	F138-I141	F138-I193	R202-G133	T203-T135	E150-S197/L198-S194	E11-H80/I81/S77-Q107
AtTIP2;1 (*Arabidopsis thaliana*)	5I32	Xray 1.18 Å	A131-L137	A131-F191	R200-H131	S201-H131	E146-S195/M196-S192	E24-H81/V82/S78-Q108
PfAQP (Plasmodium falciparum)	3C02	Xray 2.05 Å	N129-I132	N129-N187	R196-W124	D197-T126	E141-A191/L192-T188	E16-H68/L69/S65-Q95
hAQP7	6QZI	Xray 1.90 Å	P163-M166		R229-F158	D230-T160	E175-A224/I225-T221	E40-H92/M93/S89-Q119
hAQP10	6F7H	Xray 2.30 Å	A151-L154	A151-N208	R217-F146	N218-T148	Q163-L213	E27-H80/L81-Q107
GlpF (*Escherichia coli*)	1FX8	Xray 2.20 Å	N140-I143	N140-L197	R206-F135	D207-T137	E152-A201/M202-T198	E14-H66/L67/S63-Q93
AQP0 (*Ovis aries*)	2B6O	EM 1.90 Å	H122-V125	H122-Y178	R187-A117	S188-N119	E134-G182/M183-T179	E16-V67/H66/S63-Q93

Table S2. Backbone amide H/D exchange rates at four temperatures and the corresponding activation energies of unfolding (Peaks that could not be resolved in certain experiments are marked as N/A).

Seq	Res	329K (s^{-1})	Error (s^{-1})	330K (s^{-1})	Error (s^{-1})	331K (s^{-1})	Error (s^{-1})	332K (s^{-1})	Error (s^{-1})	Activation Energy (kJ/mol)	Error (kJ/mol)
16	ALA	3.54E-05	8.87E-06	1.18E-04	1.90E-05	1.88E-04	3.72E-05	4.50E-04	6.72E-05	749.90	82.96
17	GLU	6.68E-05	2.25E-05	8.99E-05	1.50E-05	1.89E-04	4.10E-05	4.57E-04	1.14E-04	575.88	77.56
18	PHE	9.05E-05	3.65E-05	1.25E-04	2.85E-05	2.08E-04	6.03E-05	5.45E-04	1.69E-04	524.22	87.67
19	LEU	2.57E-04	7.71E-05	1.10E-04	2.47E-05	2.24E-04	8.92E-05	6.34E-04	1.92E-04	789.52	65.18
21	THR	6.09E-05	1.81E-05	1.40E-04	2.69E-05	1.75E-04	3.84E-05	4.50E-04	1.22E-04	574.83	76.82
23	LEU	4.55E-05	1.53E-05	1.22E-04	3.20E-05	1.74E-04	4.22E-05	4.79E-04	1.27E-04	679.48	77.19
24	PHE	6.22E-05	1.30E-05	1.27E-04	3.70E-05	1.82E-04	4.67E-05	4.23E-04	1.11E-04	556.89	51.04
25	VAL	5.04E-05	1.84E-05	1.16E-04	3.09E-05	2.65E-04	8.91E-05	5.66E-04	1.28E-04	730.40	9.53
26	PHE	1.74E-04	5.48E-05	N/A	N/A	N/A	N/A	N/A	N/A	---	---
27	ILE	5.96E-05	1.78E-05	1.23E-04	2.90E-05	1.89E-04	3.95E-05	4.57E-04	8.14E-05	590.72	50.66
29	ILE	5.35E-05	1.99E-05	1.36E-04	2.99E-05	1.80E-04	4.20E-05	4.33E-04	7.18E-05	600.44	77.91
30	GLY	5.08E-05	6.60E-06	1.07E-04	2.55E-05	1.44E-04	2.08E-05	3.89E-04	1.07E-04	594.50	75.80
31	SER	9.46E-05	2.01E-05	1.66E-04	4.26E-05	3.58E-04	4.92E-05	6.75E-04	7.26E-05	602.92	17.63
44	THR	3.73E-03	1.46E-03	1.51E-03	1.91E-04	2.12E-03	5.28E-04	2.34E-03	5.29E-04	211.88	48.83
48	ASP	---	---	---	---	---	---	3.89E-03	4.87E-04	---	---
49	ASN	---	---	---	---	---	---	4.82E-03	7.81E-04	---	---
50	VAL	---	---	---	---	---	---	4.14E-03	4.72E-04	---	---
51	LYS	---	---	---	---	---	---	2.19E-03	6.39E-04	---	---
52	VAL	1.92E-04	1.45E-05	4.32E-04	8.17E-05	6.20E-04	6.05E-05	7.99E-04	6.93E-05	443.20	57.09
53	SER	1.95E-04	2.17E-05	5.49E-04	1.29E-04	6.19E-04	1.08E-04	1.07E-03	1.09E-04	503.90	59.49
54	LEU	1.61E-04	3.40E-05	3.50E-04	1.25E-04	4.54E-04	1.15E-04	1.06E-03	2.03E-04	555.27	54.27
56	PHE	8.86E-05	3.23E-05	1.50E-04	3.73E-05	2.94E-04	3.62E-05	7.23E-04	1.62E-04	628.74	49.14
57	GLY	6.11E-05	2.32E-05	1.14E-04	2.85E-05	2.36E-04	5.76E-05	7.69E-04	2.53E-04	754.77	77.04
59	SER	8.06E-05	1.90E-05	1.39E-04	4.01E-05	2.25E-04	3.52E-05	7.97E-04	1.98E-04	682.70	104.40
60	ILE	5.45E-05	1.79E-05	1.42E-04	3.27E-05	1.86E-04	3.82E-05	5.19E-04	1.38E-04	649.88	81.18
61	ALA	6.91E-05	1.97E-05	1.07E-04	3.47E-05	1.75E-04	3.50E-05	6.18E-04	1.50E-04	643.01	121.59
62	THR	9.06E-05	2.14E-05	1.23E-04	2.75E-05	2.03E-04	4.19E-05	5.46E-04	2.27E-04	560.36	92.14
63	LEU	1.01E-04	2.11E-05	1.59E-04	2.33E-05	1.97E-04	5.39E-05	5.42E-04	1.60E-04	488.43	112.79
64	ALA	6.02E-05	2.07E-05	1.06E-04	2.47E-05	2.11E-04	4.56E-05	6.73E-04	2.20E-04	722.64	79.92
65	GLN	8.10E-05	3.12E-05	1.10E-04	3.12E-05	2.45E-04	8.83E-05	6.20E-04	2.23E-04	620.41	89.15
66	SER	1.92E-04	2.71E-05	3.44E-04	5.53E-05	5.05E-04	1.10E-04	1.25E-03	3.30E-04	524.15	57.26
67	VAL	9.30E-05	1.12E-05	1.59E-04	1.74E-05	2.51E-04	3.99E-05	9.43E-04	1.92E-04	698.93	134.24

73	ALA	8.47E-04	1.49E-04	9.35E-04	1.37E-04	1.13E-03	2.21E-04	1.82E-03	3.70E-04	227.52	56.40
74	HIS	1.27E-04	2.13E-05	1.85E-04	5.87E-05	2.98E-04	6.44E-05	1.11E-03	2.83E-04	665.63	146.72
75	LEU	9.67E-05	1.96E-05	1.86E-04	4.46E-05	3.94E-04	9.25E-05	9.36E-04	2.47E-04	691.32	32.20
76	ASN	7.24E-05	1.92E-05	1.63E-04	5.83E-05	1.44E-04	3.91E-05	5.43E-04	1.18E-04	575.28	155.61
78	ALA	5.08E-05	1.66E-05	1.08E-04	2.83E-05	2.09E-04	4.64E-05	4.58E-04	1.01E-04	659.53	14.13
79	VAL	5.62E-05	2.34E-05	1.14E-04	3.34E-05	2.53E-04	4.92E-05	8.07E-04	1.28E-04	774.30	64.06
80	THR	3.66E-05	9.96E-06	9.10E-05	1.54E-05	1.36E-04	3.45E-05	4.44E-04	1.14E-04	721.08	89.06
96	MET	7.91E-05	3.65E-05	1.32E-04	3.06E-05	2.31E-04	7.28E-05	8.38E-04	3.16E-04	687.19	111.53
98	ILE	2.82E-05	5.76E-06	1.13E-04	1.78E-05	1.91E-04	5.18E-05	5.98E-04	1.64E-04	885.98	90.62
99	ILE	3.62E-05	1.01E-05	1.22E-04	2.68E-05	1.66E-04	3.11E-05	3.27E-04	8.96E-05	637.06	104.18
100	ALA	1.91E-05	9.94E-06	1.09E-04	2.73E-05	1.92E-04	6.32E-05	4.60E-04	8.28E-05	981.97	148.47
101	GLN	7.94E-05	2.54E-05	1.04E-04	1.88E-05	2.17E-04	4.27E-05	6.17E-04	1.13E-04	625.93	114.00
102	CYS	5.94E-05	2.04E-05	1.16E-04	2.28E-05	1.69E-04	4.70E-05	5.30E-04	1.40E-04	630.39	97.74
104	GLY	3.99E-05	1.60E-05	1.06E-04	2.41E-05	2.48E-04	6.49E-05	3.65E-04	1.44E-04	679.25	77.85
105	ALA	4.84E-05	7.58E-06	1.07E-04	2.75E-05	1.98E-04	5.34E-05	4.48E-04	1.12E-04	666.04	18.78
106	ILE	8.53E-05	4.46E-05	1.05E-04	2.78E-05	3.72E-04	8.74E-05	7.78E-04	2.11E-04	683.21	116.58
107	VAL	4.38E-05	1.62E-05	9.47E-05	1.83E-05	1.93E-04	5.06E-05	4.44E-04	6.23E-05	691.30	14.76
109	THR	6.34E-05	1.89E-05	9.81E-05	1.93E-05	1.84E-04	3.97E-05	4.83E-04	8.01E-05	655.24	77.07
113	SER	6.98E-04	1.82E-04	8.14E-04	1.48E-04	1.15E-03	2.65E-04	1.75E-03	2.64E-04	303.93	37.32
115	ILE	7.65E-04	2.70E-04	4.32E-04	1.10E-04	1.79E-03	2.29E-04	1.38E-03	1.72E-04	---	---
119	LEU	---	---	---	---	---	---	5.51E-03	6.56E-04	---	---
120	THR	---	---	---	---	---	---	6.94E-03	1.38E-03	---	---
121	GLY	---	---	---	---	---	---	5.96E-03	6.48E-04	---	---
122	ASN	---	---	---	---	---	---	1.06E-02	1.99E-03	---	---
124	LEU	---	---	---	---	---	---	6.31E-03	9.43E-04	---	---
125	GLY	---	---	---	---	---	---	6.05E-03	1.10E-03	---	---
126	ARG	---	---	---	---	---	---	4.85E-03	9.35E-04	---	---
127	ASN	---	---	---	---	---	---	4.30E-03	4.90E-04	---	---
128	ASP	---	---	---	---	---	---	7.46E-03	4.81E-04	---	---
129	LEU	---	---	---	---	---	---	4.70E-03	7.74E-04	---	---
130	ALA	9.08E-04	5.37E-05	1.81E-03	1.87E-04	3.85E-03	3.86E-04	3.69E-03	3.46E-04	474.38	96.97
131	ASP	---	---	---	---	---	---	6.89E-03	1.24E-03	---	---
132	GLY	---	---	---	---	---	---	6.09E-03	9.20E-04	---	---
133	VAL	9.50E-04	9.74E-05	1.70E-03	1.08E-04	2.34E-03	2.46E-04	2.62E-03	3.44E-04	304.13	62.04
134	ASN	4.82E-03	6.83E-04	3.90E-03	3.57E-04	8.02E-03	9.32E-04	4.31E-03	6.29E-04	---	---
135	SER	---	---	---	---	---	---	5.99E-03	1.16E-03	---	---
136	GLY	---	---	---	---	---	---	4.06E-03	1.19E-03	---	---
140	GLY	1.33E-04	1.81E-05	2.50E-04	4.06E-05	4.03E-04	7.63E-05	9.54E-04	1.14E-04	591.48	37.40
141	ILE	5.06E-05	1.23E-05	1.04E-04	2.35E-05	2.23E-04	3.40E-05	5.34E-04	1.21E-04	712.89	21.88
142	GLU	8.18E-05	2.66E-05	1.08E-04	3.47E-05	2.15E-04	4.99E-05	4.97E-04	6.45E-05	598.54	71.01

143	ILE	5.36E-05	1.60E-05	1.47E-04	4.02E-05	1.91E-04	4.70E-05	3.64E-04	1.19E-04	548.67	84.21
144	ILE	1.10E-04	3.17E-05	8.28E-05	1.71E-05	1.52E-04	3.22E-05	5.68E-04	2.09E-04	522.08	207.47
145	GLY	5.29E-05	8.65E-06	9.78E-05	2.41E-05	1.80E-04	5.13E-05	4.82E-04	8.15E-05	665.19	43.75
146	THR	3.55E-05	1.45E-05	1.15E-04	3.01E-05	1.93E-04	3.73E-05	4.55E-04	1.20E-04	758.05	73.55
147	LEU	4.84E-05	1.04E-05	1.32E-04	2.84E-05	1.63E-04	3.99E-05	3.70E-04	9.80E-05	574.19	90.34
148	GLN	7.47E-05	1.98E-05	1.18E-04	3.78E-05	1.56E-04	3.04E-05	4.88E-04	1.40E-04	550.36	107.18
151	LEU	6.26E-05	1.15E-05	1.33E-04	3.96E-05	2.27E-04	8.93E-05	6.17E-04	1.00E-04	690.08	30.28
152	CYS	4.66E-05	1.65E-05	1.07E-04	1.93E-05	1.56E-04	3.50E-05	4.47E-04	9.54E-05	649.98	70.83
153	VAL	5.17E-05	9.79E-06	1.15E-04	2.52E-05	1.71E-04	3.86E-05	6.16E-04	1.71E-04	684.51	103.99
155	ALA	7.12E-05	1.50E-05	9.42E-05	3.41E-05	1.89E-04	4.71E-05	5.01E-04	1.71E-04	625.70	102.49
156	THR	---	---	1.33E-04	2.34E-05	2.00E-04	6.00E-05	1.79E-03	4.94E-04	---	---
157	THR	---	---	---	---	---	---	5.07E-03	9.40E-04	---	---
158	ASP	---	---	---	---	---	---	6.26E-03	1.17E-03	---	---
159	ARG	---	---	---	---	---	---	7.52E-03	2.27E-03	---	---
167	SER	---	---	---	---	---	---	6.85E-03	9.19E-04	---	---
168	ALA	---	---	---	---	---	---	6.32E-03	9.74E-04	---	---
172	ILE	1.19E-03	1.89E-04	1.67E-03	3.94E-04	2.35E-03	3.79E-04	2.54E-03	3.00E-04	228.85	38.64
173	GLY	2.18E-04	2.65E-05	2.98E-04	4.26E-05	4.66E-04	7.62E-05	1.01E-03	1.47E-04	462.01	71.32
174	LEU	8.73E-05	2.24E-05	N/A	N/A	1.65E-04	4.75E-05	4.30E-04	1.30E-04	461.24	148.90
175	SER	6.19E-05	1.74E-05	N/A	N/A	N/A	N/A	N/A	N/A	---	---
176	VAL	3.80E-05	1.35E-05	1.31E-04	3.14E-05	1.94E-04	4.06E-05	5.07E-04	1.05E-04	758.08	95.49
177	ALA	4.17E-05	9.66E-06	1.51E-04	3.47E-05	2.80E-04	7.85E-05	5.39E-04	1.84E-04	743.91	94.41
178	LEU	7.32E-05	2.51E-05	1.24E-04	2.96E-05	1.96E-04	3.99E-05	6.21E-04	1.52E-04	618.69	93.04
179	GLY	1.36E-04	4.61E-05	1.40E-04	4.95E-05	1.95E-04	5.91E-05	4.06E-04	1.16E-04	479.05	103.09
180	HIS	6.45E-05	1.42E-05	1.24E-04	2.89E-05	2.51E-04	4.32E-05	4.69E-04	9.45E-05	604.90	8.77
181	LEU	5.89E-05	1.54E-05	1.07E-04	2.57E-05	1.65E-04	2.14E-05	2.92E-04	3.33E-05	476.17	18.81
182	LEU	5.13E-05	1.95E-05	1.23E-04	3.13E-05	2.38E-04	5.17E-05	4.27E-04	8.46E-05	649.28	40.61
183	ALA	5.24E-05	1.16E-05	1.25E-04	3.95E-05	2.20E-04	4.59E-05	4.32E-04	8.46E-05	624.10	42.98
184	ILE	1.76E-04	2.79E-05	2.69E-04	4.14E-05	4.90E-04	8.13E-05	1.00E-03	1.54E-04	527.19	42.77
185	ASP	---	---	---	---	---	---	3.77E-03	6.89E-04	---	---
186	TYR	7.83E-04	2.49E-05	9.60E-04	8.40E-05	1.61E-03	2.53E-04	1.78E-03	2.31E-04	255.78	32.63
187	THR	4.88E-04	8.46E-05	5.90E-04	9.89E-05	1.34E-03	1.45E-04	1.99E-03	2.81E-04	465.92	74.54
188	GLY	9.79E-04	1.48E-04	1.79E-03	1.54E-04	3.18E-03	3.06E-04	3.42E-03	5.16E-04	389.42	72.25
189	CYS	2.27E-03	2.35E-04	3.26E-03	4.32E-04	5.52E-03	7.60E-04	3.54E-03	4.52E-04	130.26	68.15
190	GLY	---	---	---	---	---	---	3.98E-03	2.65E-04	---	---
191	ILE	---	---	---	---	---	---	5.03E-03	4.76E-04	---	---
192	ASN	---	---	---	---	---	---	4.40E-03	9.74E-04	---	---
194	ALA	6.15E-05	1.34E-05	1.38E-04	2.93E-05	2.14E-04	4.63E-05	4.79E-04	1.07E-04	600.54	46.60
195	ARG	6.74E-05	1.20E-05	1.34E-04	3.85E-05	2.90E-04	6.87E-05	4.50E-04	7.72E-05	592.55	46.48
196	SER	3.68E-04	8.19E-05	4.68E-04	5.22E-05	8.94E-04	5.11E-05	1.33E-03	2.57E-04	397.80	37.92

197	PHE	9.32E-05	1.94E-05	1.09E-04	1.86E-05	2.52E-04	4.03E-05	6.79E-04	1.11E-04	603.90	123.83
199	SER	1.76E-04	3.38E-05	N/A	N/A	N/A	N/A	N/A	N/A	---	---
200	ALA	6.50E-05	1.05E-05	N/A	N/A	N/A	N/A	N/A	N/A	---	---
205	ASN	---	---	2.59E-03	2.93E-04	2.28E-03	5.29E-04	3.17E-03	5.35E-04	---	---
206	PHE	---	---	---	---	---	---	3.33E-03	8.12E-04	---	---
210	TRP	---	---	---	---	---	---	5.78E-03	1.63E-03	---	---
211	ILE	6.56E-04	1.74E-04	6.85E-04	1.03E-04	1.24E-03	1.82E-04	1.07E-03	2.34E-04	175.34	71.47
213	TRP	4.60E-05	1.75E-05	9.77E-05	2.29E-05	1.65E-04	3.11E-05	3.27E-04	1.01E-04	586.74	22.78
214	VAL	2.51E-05	6.93E-06	1.10E-04	2.59E-05	1.99E-04	2.97E-05	4.61E-04	7.32E-05	813.09	101.04
217	PHE	7.28E-05	3.18E-05	9.90E-05	3.62E-05	1.65E-04	3.36E-05	5.82E-04	1.44E-04	592.82	131.97
219	GLY	4.03E-05	1.98E-05	1.50E-04	5.44E-05	1.62E-04	5.27E-05	4.47E-04	1.84E-04	680.20	133.34
220	GLY	4.48E-05	1.56E-05	1.50E-04	4.29E-05	2.25E-04	6.19E-05	5.66E-04	1.43E-04	735.89	93.75
221	ALA	8.06E-05	2.09E-05	1.22E-04	2.70E-05	1.79E-04	5.10E-05	4.82E-04	8.77E-05	555.22	81.36
222	LEU	5.23E-05	1.31E-05	9.35E-05	2.03E-05	1.84E-04	3.44E-05	5.24E-04	1.54E-04	697.67	64.61
223	ALA	6.57E-05	1.98E-05	1.25E-04	3.45E-05	2.30E-04	4.05E-05	4.63E-04	1.18E-04	588.61	10.87
224	VAL	6.84E-05	1.39E-05	1.00E-04	3.01E-05	2.01E-04	5.00E-05	4.44E-04	6.90E-05	576.57	47.78
226	ILE	2.30E-05	1.09E-05	1.18E-04	2.88E-05	2.82E-04	9.17E-05	6.52E-04	2.12E-04	914.99	130.29

Table S3. Side chain amide H/D exchange rates measured at four temperatures.

Res	Atom	329K (s^{-1})	Error (s^{-1})	330K (s^{-1})	Error (s^{-1})	331K (s^{-1})	Error (s^{-1})	332K (s^{-1})	Error (s^{-1})	Activation Energy (kJ/mol)	Error (kJ/mol)
N127	ND2	1.57E-03	2.70E-04	8.92E-04	1.95E-04	2.11E-03	3.87E-04	9.88E-04	1.97E-04	---	---
R195	NH1	---	---	6.81E-04	3.48E-04	2.85E-03	9.18E-04	4.46E-04	8.03E-05	---	---
R195	NH2	---	---	2.67E-03	8.76E-04	1.49E-03	3.15E-04	5.94E-04	1.32E-04	---	---
R195	NE	3.03E-03	1.39E-03	4.26E-03	1.27E-03	1.00E-02	2.99E-03	1.35E-03	2.97E-04	---	---
H180	NE2	---	---	---	---	8.24E-03	1.56E-03	2.45E-03	2.84E-04	---	---
N205	ND2	---	---	9.46E-04	4.16E-04	2.26E-03	5.05E-04	8.75E-04	1.33E-04	---	---
N122	ND2	---	---	1.79E-03	8.69E-04	3.88E-03	7.38E-04	1.59E-03	5.03E-04	---	---
Q137	NE2	---	---	1.46E-03	6.51E-04	4.95E-03	7.30E-04	1.04E-03	2.34E-04	---	---
